# Isotope Probing of the UDP‐Apiose/UDP‐Xylose Synthase Reaction: Evidence of a Mechanism via a Coupled Oxidation and Aldol Cleavage

**DOI:** 10.1002/anie.201609288

**Published:** 2017-01-19

**Authors:** Thomas Eixelsberger, Doroteja Horvat, Alexander Gutmann, Hansjörg Weber, Bernd Nidetzky

**Affiliations:** ^1^ Institute of Biotechnology and Biochemical EngineeringGraz University of TechnologyNAWI GrazPetersgasse 128010GrazAustria; ^2^ Institute of Organic ChemistryGraz University of TechnologyNAWI GrazStremayrgasse 98010GrazAustria; ^3^ Austrian Centre of Industrial Biotechnology (acib)Petersgasse 148010GrazAustria

**Keywords:** aldol reactions, carbohydrates, enzyme catalysis, reaction mechanism, ring contraction

## Abstract

The C‐branched sugar d‐apiose (Api) is essential for plant cell‐wall development. An enzyme‐catalyzed decarboxylation/pyranoside ring‐contraction reaction leads from UDP‐α‐d‐glucuronic acid (UDP‐GlcA) to the Api precursor UDP‐α‐d‐apiose (UDP‐Api). We examined the mechanism of UDP‐Api/UDP‐α‐d‐xylose synthase (UAXS) with site‐selectively ^2^H‐labeled and deoxygenated substrates. The analogue UDP‐2‐deoxy‐GlcA, which prevents C‐2/C‐3 aldol cleavage as the plausible initiating step of pyranoside‐to‐furanoside conversion, did not give the corresponding Api product. Kinetic isotope effects (KIEs) support an UAXS mechanism in which substrate oxidation by enzyme‐NAD^+^ and retro‐aldol sugar ring‐opening occur coupled in a single rate‐limiting step leading to decarboxylation. Rearrangement and ring‐contracting aldol addition in an open‐chain intermediate then give the UDP‐Api aldehyde, which is intercepted via reduction by enzyme‐NADH.

Uridine 5′‐diphosphate (UDP)‐α‐d‐apiose (**1**) is the precursor of the C‐branched pentose d‐apiose [3‐*C*‐(hydroxymethyl)‐d‐*glycero*‐tetrose; **2**].^1^ Compound **2** is present in the cell‐wall polysaccharides rhamnogalacturonan II and apiogalacturonan, as well as in various secondary metabolites in plants.^1^, ^2^, ^3^, ^4^ Sugar nucleotide **1** is derived from UDP‐α‐d‐glucuronic acid (**3**) in a decarboxylation/pyranoside ring‐contraction reaction catalyzed by UDP‐α‐d‐apiose/UDP‐α‐d‐xylose synthase (UAXS).^5^ The proposed mechanism of this chemically intriguing biotransformation (Scheme 1) involves nicotinamide adenine dinucleotide (NAD^+^)‐assisted oxidation at C‐4 of substrate **3** and subsequent decarboxylation to give UDP‐β‐l‐*threo*‐pentopyranosid‐4‐ulose (**4**).^5^, ^6^, ^7^ Carbon skeleton rearrangement (**4**→**5**→**6**) then occurs probably via a retro‐aldol/aldol reaction,^6^ and reduction of UDP‐α‐d‐apiose 3′‐aldehyde (**6**) by enzyme‐NADH gives **1**.^5^, ^6^, ^7^ The alternative reaction product, UDP‐α‐d‐xylose (**7**), is derived from **4**, also by NADH‐dependent reduction. UDP‐α‐d‐xylose synthase (UXS) is structurally and mechanistically related to UAXS, but lacks the ability to catalyze the pyranoside‐into‐furanoside conversion.^8^ A plausible point of divergence in the proposed pathways of UAXS and UXS is therefore intermediate **4**.

**Scheme 1:**
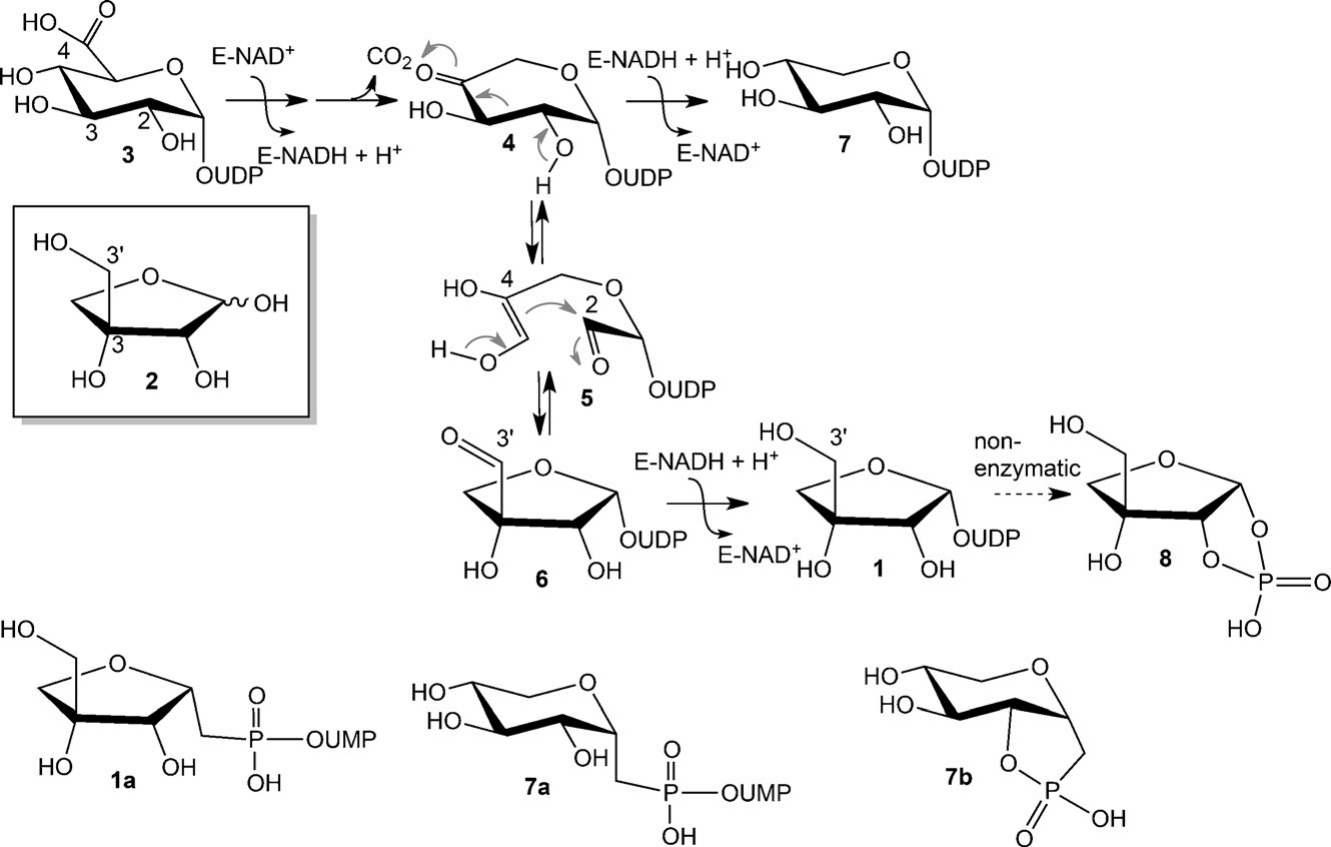
The proposed mechanism of UAXS; E=enzyme.^5^, ^6^, ^7^

Its widespread acceptance in the literature notwithstanding,^5^, ^6^, ^7^ the mechanism of Scheme 1 raises disquiet in that it requires UAXS to recognize **4** equally for aldol ring cleavage and for reduction by NADH. How the enzyme distinguishes between these possibilities is not clear. Moreover, there is only indirect evidence in support of the retro‐aldol/aldol route of conversion of **4** into **6**. A 2‐deoxy‐2‐fluoro analogue of **3**, rendering impossible the C‐2/C‐3 aldol cleavage in a corresponding 2‐fluoro derivative of **4**, was completely unreactive with UAXS.6a A chemically stable phosphonate analogue of **1** (Scheme 1, **1 a**) was converted by UAXS into the corresponding xylosyl compound. A xylose cyclic phosphonate (**7 b**) instead of the expected product **7 a** (Scheme 1) was formed. This was interpreted to involve an enzymatically deprotonated C‐2 hydroxy group, which would also be involved in the “native” retro‐aldol conversion of **4**.6b The current study was performed to re‐investigate the catalytic steps for conversion of substrate **3** into **1** and **7**. Evidence supporting an updated mechanism, involving retro‐aldol ring opening prior to the decarboxylation, is presented.

Purified UAXS from *Arabidopsis thaliana* recombinantly expressed in *Escherichia coli* was used (Figure S1 in the Supporting Information). Reactions were performed at pH 8.5 because the enzyme activity was highest (Table S1, Supporting Information) and the ratio of product **1** to **7** maximized under these conditions. No intermediary products (e.g., **4**)6b, 7 were released. Under the conditions used, α‐d‐apiofuranosyl‐1,2‐cyclic phosphate (**8**; Scheme 1) was spontaneously formed from **1**.^5^, ^7^ Product **1** was therefore always detected as **8**. Site‐selectively ^13^C‐ or ^2^H‐labeled analogues of substrate **3** were synthesized from the corresponding isotopically labeled d‐glucoses (Scheme S1, Figure S2–S11).9a Unlabeled **3** was prepared identically and used as a reference. A 2‐deoxy analogue of **3** was synthesized from 1,5‐anhydro‐2‐deoxy‐d‐*arabino*‐hex‐1‐enitol via 2‐deoxy‐d‐glucose‐1‐phosphate, exploiting the reaction of cellobiose phosphorylase (Scheme S2, Figure S12–S18).9b Each compound was isolated and its identity confirmed by ^1^H and ^13^C NMR spectroscopy. Purity was furthermore determined by capillary electrophoresis and HPLC.

In situ NMR analysis of enzymatic reactions of [2‐^13^ *C*]‐**3** and [3‐^13^ *C*]‐**3** demonstrated rearrangement of the carbon skeleton on formation of **1** as expected from Scheme 1. Despite being confirmatory mainly,^5^, ^6^, ^7^ the evidence was nonetheless crucial for it enabled the precise assignment of all the ^13^C signals from substrate and products (Figure S19–S24). Reaction of unlabeled **3** in D_2_O resulted in a single deuterium from solvent to be incorporated at C‐4 of **1** and C‐5 of **7** as result of the decarboxylation, consistent with literature.5d,5e Reactions of [3‐^2^H]‐**3** and [4‐^2^H]‐**3** both gave [3′‐^2^H]‐**1**, as expected from Scheme 1,5e however with clearly distinct C^2^H^1^H groups at position 3′ (Figure 1 A, panels a,b; Figure S25). Because reduction of **6** by the hydrogen or deuterium abstracted from C‐4 is stereospecific,5d,5e the absolute configuration at C‐3′ in **1** will be opposite in the two conversions. Irrespective of whether reaction of unlabeled **3** was examined in D_2_O or reaction of [3‐^2^H]‐**3** in water, no ^1^H/^2^H exchange was observed at position 3′ (Figure 1 A, panels c,d; Figure S25) The result was important mechanistically for it validated the determination of kinetic isotope effects (KIEs) through the deuteration at C‐3 of **3**. Studying the UAXS from parsley or duckweed, however, with a relatively complicated and indirect procedure of product analysis, other authors reported uptake of 0.5 mol ^3^H mol^−1^ in **1** at C‐3′.5b,5e We rule out a similar exchange with protons from solvent in the enzymatic reaction under the conditions we used.

**Figure 1:**
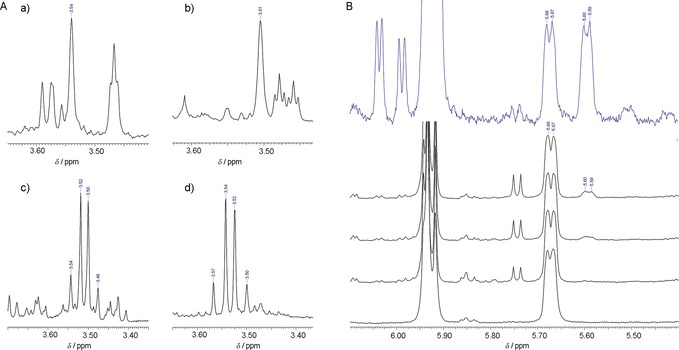
A) Formation of product **1** (detected as **8**) by UAXS. a),b) ^1^H NMR signal of the 3′‐H in **8** obtained from [3‐^2^H]‐**3** (a) and [4‐^2^H]‐**3** (b). Both reactions were carried out in H_2_O (pH 8.5). c),d) ^1^H NMR signal of 3′‐H in **8** obtained from unlabeled **3** in D_2_O (c) and H_2_O (d). In panels (a)–(d), the product was analyzed directly from the reaction mixture. Enzyme: 20 μm; substrate **3**: 2 mm; pH(D)=8.5. B) Formation of 2‐deoxy‐**7** by UAXS and UXS. In blue: the ^1^H NMR spectrum of a reaction mixture of 2‐deoxy‐**3** (δ=5.67 ppm, 5.68 ppm) converted partially into 2‐deoxy‐**7** (δ=5.59 ppm, 5.60 ppm) by UXS. The black stack plot shows an in situ ^1^H NMR experiment of the conversion of 2‐deoxy‐**3** (δ=5.67 ppm, 5.68 ppm) by UAXS. It shows that 2‐deoxy‐**7** (δ=5.59 ppm, 5.60 ppm) is formed in small amounts. UXS: 20 μm; UAXS: 100 μm; substrate 2‐deoxy‐**3**: 2 mm. Reactions were performed in D_2_O (pD=8.5) for 2 h (UXS) and over 12 h with spectra recording in 2 h intervals (UAXS).

2‐Deoxy‐**3** was examined as a substrate of UAXS for it prevents the C‐2/C‐3 retro‐aldol cleavage to initiate pyranoside‐into‐furanoside ring conversion. Compared to 2‐deoxy‐2‐fluoro‐**3** used earlier with the same rationale,6a 2‐deoxy‐**3** features only weak electronic perturbation at the position 2, thus rendering it a mechanistic probe of the enzyme in its own right. Because preliminary experiments suggested UAXS to be inactive towards 2‐deoxy‐**3**, we also tested UXS and demonstrated enzymatic conversion to give 2‐deoxy‐**7** as the product (Figure 1 B, Figure S26–S28). Noticing that UAXS might be inhibited by UDP/UMP released from 2‐deoxy‐**3** due to decomposition, we re‐examined UAXS under addition of alkaline phosphatase to hydrolyze the nucleosides present. Synthesis of a tiny amount of 2‐deoxy‐**7** was shown under these conditions (Figure 1 B).

No evidence of 2‐deoxy‐**1** was found, as expected. However, while UXS exhibited substantial activity with 2‐deoxy‐**3** (Figure 1 B; Figure S26), the UAXS activity was almost completely destroyed (≤0.1 % of activity with **3**) on substituting the 2‐OH in the substrate by a hydrogen atom. These results are important mechanistically showing that 2‐deoxy‐**3** was fully competent to undergo oxidative decarboxylation in the UXS‐type reaction, plausibly via 2‐deoxy‐**4**. Assuming intermediate **4** to be the point of divergence in the UAXS and UXS reaction paths, the huge difference in activity of the two enzymes in forming 2‐deoxy‐**7** was somewhat counterintuitive. Note that only a small change in the reaction conditions (e.g. pH, ion type and concentration) is sufficient to shift the distribution of UAXS reaction products to favor **7**, while at the same time the overall activity is just moderately affected (data not shown; see also Ref. 5f). We therefore hypothesized that the pyranoside ring‐opening characteristic of UAXS might not be as clearly decoupled from the oxidation–decarboxylation common to both enzymatic conversions as the mechanistic proposal of Scheme 1 assumes. Intermediate **4** might be formed through a different path in UAXS than in UXS, and we designed a KIE study to address the problem. It will become clear below that the minute conversion of 2‐deoxy‐**3** by UAXS is probably a relic of UXS‐type activity in this enzyme, which considering the relatedness of UAXS and UXS at the level of amino acid sequence^10^ is not a complete surprise.

We determined the effect of [4‐^2^H] in **3** on the rate of substrate consumption (*V*_S_). Direct comparison of *V*_S_ with *protio* and *deuterio* substrates at saturating concentration gave a large KIE of 2.72±0.20 (*N*=5). The KIE measured by intermolecular competition was also high and its value (2.47±0.43) was similar to the KIE on *k*_cat_ (Table S2). Contrary to the directly determined KIE, which influences the catalytic constant (*k*_cat_), the KIE from the competition experiment necessarily influences the second‐order rate constant (*k*_cat_/*K*_mS_).^11^ Scheme 2 is used for interpretation of the data.

**Scheme 2:**
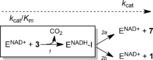
A minimal kinetic mechanism of UAXS is shown. “I” represents an enzyme‐bound intermediate suggested to be the acyclic form **5**. A primary deuterium KIE could arise in steps 1, 2 a and 2 b. The sequence of reaction steps included in *k*_cat_/*K*_m_ is shown in the box.

Whereas *k*_cat_ involves all the unimolecular steps of the reaction, *k*_cat_/*K*_mS_ includes only those steps up to the first irreversible step, which in UAXS is the decarboxylation. The large KIE on *k*_cat_/*K*_mS_ implies that hydride abstraction from substrate is partly rate limiting for the steps covered by *k*_cat_/*K*_mS_. The UAXS reaction involves three isotope‐sensitive steps (Scheme 1 and Scheme 2) and each could contribute to the KIE on *k*_cat_. We measured under rapid‐mixing conditions by absorbance at 340 nm the reduction of enzyme‐bound NAD^+^. NADH did not accumulate in detectable amounts, which it would if the two hydride transfers from NADH were limiting for *k*_cat_. This result together with the evidence that *k*_cat_/*K*_mS_ and *k*_cat_ were affected by a similar KIE, identified hydride abstraction from substrate to NAD^+^ as major rate‐determining step of the UAXS reaction. Although hydride transfers from enzyme‐NADH were not slow steps of the overall reaction, the ratio of the products **1** and **7** was nonetheless affected by [4‐^2^H] in **3**. The ratio changed from a value of 1.84 with undeuterated **3** to a lower value of 1.48 with [4‐^2^H]‐**3** (Table S3). Therefore, this suggested a larger deuterium KIE on reduction of **6** than on reduction of **4**. Moreover, the result demonstrated the ability of UAXS to reversibly interconvert intermediates **4** and **6**, in good agreement with the results of Liu and co‐workers,6b and to rapidly equilibrate their enzyme‐bound forms.

Next, we determined in intermolecular competition experiments the effect from [3‐^2^H] in **3** on *k*_cat_/*K*_mS_. We considered that a KIE would be a secondary one, arising from hybridization change at C‐3 during sugar ring opening and closure. And additionally, because substrate oxidation was a rate‐limiting step, a KIE different from unity would be possible only in the case that the reaction step(s) affecting C‐3 happened within the rate‐determining cascade of oxidation and decarboxylation. Starting from a substrate composed initially of roughly equal amounts of **3** and [3‐^2^H]‐**3**, we determined by NMR spectroscopy at three different levels of conversion the ^1^H/^2^H isotope ratio at C‐3 in the remaining substrate. A KIE of 1.20±0.03 was obtained (Table S4), indicating that [3‐^2^H]‐**3** reacted significantly more slowly than **3**. The observed effect was in the upper region of the KIEs previously reported from enzyme‐catalyzed aldol/retro‐aldol reactions.^12^ It would be consistent with a fully developed secondary KIE resulting from complete sp^3^→sp^2^ hybridization change between the ground state and the rate‐limiting transition state of a retro‐aldol carbon–carbon bond cleavage for enzymatic pyranosyl ring opening. It may be noted that secondary deuterium KIEs of similar magnitude were observed in other enzymes catalyzing aldol reactions.^12^ Additionally we measured the ^1^H/^2^H isotope ratio at C‐3′ in **1** and C‐3 in **7** at approximately 50 % conversion and found its value of approximately 1.2 to be the same in both products and to reflect exactly the corresponding isotope ratio (i.e. 3‐^2^H/3‐^1^H) in the residual substrate (Table S5). These results indicate that reaction steps after the decarboxylation involving hybridization change at C‐3, namely the reversible aldol addition to give **4** and the reduction of **6**, did not have a significant secondary KIE. Had there been a KIE in one of these steps, the isotope ratio in **1** and **7** would not have been the same, and would also have been different from that in the **3** converted.

Taken together, the primary and secondary KIEs also suggest a relative timing of substrate oxidation and ring opening. A distinctly slow retro‐aldol reaction occurring after the oxidation at C‐4 would likely make the hydride transfer to NAD^+^ come to equilibrium. This scenario is inconsistent with the large primary KIEs observed. Therefore, a concerted transformation is supported in which hydride abstraction and ring opening take place coupled one to another in a single rate‐determining reaction step (Scheme 3). Following decarboxylation, ring closure would thus yield intermediates **4** and **6**, probably in rapid equilibrium, which are then reduced to products **7** and **1**, respectively.^13^ This way of product formation seems attractive as it avoids the dual use of intermediate **4** as substrate for enzymatic aldol cleavage and for NADH‐dependent reduction. Selective stabilization of the acyclic intermediate after the decarboxylation could be a catalytic strategy of UAXS to facilitate rapid interconversion of the more stable cyclic forms **4** (in particular) and **6**. In this mechanistic scenario, formation of 2‐deoxy‐**7** from 2‐deoxy‐**3** in the absence of ring opening is considered a minor side activity of UAXS.

**Scheme 3:**
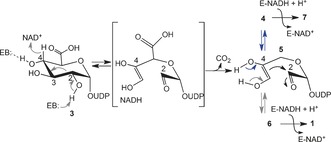
Updated mechanistic proposal for UAXS. “EB” indicates an enzyme base in the active site.

In summary, KIE and substrate analogue studies suggest an updated UAXS mechanism (Scheme 3). Aldol cleavage for pyranosyl ring opening is proposed to occur early in the reaction, concerted with the oxidation at C‐4. The reactions of UAXS and UXS would not, therefore, proceed on the same path up to intermediate **4**.^5^, ^6^, ^7^ Formation of the two UAXS products **1** and **7** involves reduction by enzyme‐NADH at distinct “exit points” of the aldol/retro‐aldol rearrangement cycle in rapid equilibrium. The UAXS mechanism, although highly specialized, possesses fundamental significance, as other C‐branched carbohydrates, such as l‐streptose (3‐*C*‐formyl‐5‐deoxy‐l‐lyxose),^14^ l‐dihydrostreptose (3‐*C*‐hydroxymethyl‐5‐deoxy‐l‐lyxose),^14^ d‐hamamelose (2‐*C*‐hydroxymethyl‐d‐ribose),^1^ and aceric acid (3‐*C*‐carboxy‐5‐deoxy‐d‐xylose)^1^ might be formed via mechanistically similar transformations.

## Conflict of interest

The authors declare no conflict of interest.

## Supporting information

As a service to our authors and readers, this journal provides supporting information supplied by the authors. Such materials are peer reviewed and may be re‐organized for online delivery, but are not copy‐edited or typeset. Technical support issues arising from supporting information (other than missing files) should be addressed to the authors.

Supplementary

## References

[1] M. Picmanová , B. L. Møller , Glycobiology 2016, 26, 430–442. 26848180 10.1093/glycob/cww012

[2] M. Mølhøj , R. Verma , W.-D. Reiter , Plant J. 2003, 35, 693–703. 12969423 10.1046/j.1365-313x.2003.01841.x

[3] M. McNeil , A. G. Darvill , S. C. Fry , P. Albersheim , Annu. Rev. Biochem. 1984, 53, 625–663. 6383202 10.1146/annurev.bi.53.070184.003205

[4a] M. A. O'Neill , S. Eberhard , P. Albersheim , A. G. Darvill , Science 2001, 294, 846–849; 11679668 10.1126/science.1062319

[4b] J. W. Ahn , R. Verma , M. Kim , J. Y. Lee , Y. K. Kim , J. W. Bang , W.-D. Reiter , H. S. Pai , J. Biol. Chem. 2006, 281, 13708–13716. 16549428 10.1074/jbc.M512403200

[5a] J. M. Picken , J. Mendicino , J. Biol. Chem. 1967, 242, 1629–1634; 4290249

[5b] J. Mendicino , H. Abou-Issa , Biochim. Biophys. Acta Enzymol. 1974, 364, 159–172; 10.1016/0005-2744(74)90143-0 4373069

[5c] H. Sandermann , G. T. Tisue , H. Grisebach , Biochim. Biophys. Acta Gen. Subj. 1968, 165, 550–552; 10.1016/0304-4165(68)90239-0 5737946

[5d] W. J. Kelleher , H. Grisebach , Eur. J. Biochem. 1971, 23, 136–142; 5127378 10.1111/j.1432-1033.1971.tb01600.x

[5e] D. Baron , H. Grisebach , Eur. J. Biochem. 1973, 38, 153–159;

[5f] C. Gebb , D. Baron , H. Grisebach , Eur. J. Biochem. 1975, 54, 493–498; 240687 10.1111/j.1432-1033.1975.tb04161.x

[5g] W. J. Kelleher , D. Baron , R. Ortmann , H. Grisebach , FEBS Lett. 1972, 22, 203–204; 11946597 10.1016/0014-5793(72)80045-0

[5h] D. Baron , E. Wellmann , H. Grisebach , Biochim. Biophys. Acta Enzymol. 1972, 258, 310–318; 10.1016/0005-2744(72)90988-6 4333589

[5i] S. Yin , J. Q. Kong , Plant Cell Rep. 2016, 35, 2304–2321. 10.1007/s00299-016-2044-5 27591771

[6a] S.-h. Choi , M. W. Ruszczycky , H. Zhang , H.-w. Liu , Chem. Commun. 2011, 47, 10130–10132; 10.1039/c1cc13140k 21826368

[6b] S.-h. Choi , S. O. Mansoorabadi , Y.-n. Liu , T.-C. Chien , H.-w. Liu , J. Am. Chem. Soc. 2012, 134, 13946–13949. 22830643 10.1021/ja305322x PMC3454503

[7a] P. Guyett , J. Glushka , X. Gu , M. Bar-Peled , Carbohydr. Res. 2009, 344, 1072–1078; 19375693 10.1016/j.carres.2009.03.026 PMC4000172

[7b] J. Smith , Y. Yang , S. Levy , O. O. Adelusi , M. G. Hahn , M. A. O'Neill , M. Bar-Peled , J. Biol. Chem. 2016, 291, 21434-21447. 27551039 10.1074/jbc.M116.749069 PMC5076816

[8a] X. M. He , H.-w. Liu , Annu. Rev. Biochem. 2002, 71, 701–754; 12045109 10.1146/annurev.biochem.71.110601.135339

[8b] C. J. Thibodeaux , C. E. Melançon III , H.-w. Liu , Angew. Chem. Int. Ed. 2008, 47, 9814–9859; 10.1002/anie.200801204 PMC2796923 19058170

[8c] M. E. Tanner , Curr. Opin. Chem. Biol. 2008, 12, 532–538; 18625333 10.1016/j.cbpa.2008.06.016

[8d] M. Bar-Peled , M. A. O'Neill , Annu. Rev. Plant Biol. 2011, 62, 127–155; 21370975 10.1146/annurev-arplant-042110-103918

[8e] T. Eixelsberger , S. Sykora , S. Egger , M. Brunsteiner , K. L. Kavanagh , U. Oppermann , L. Brecker , B. Nidetzky , J. Biol. Chem. 2012, 287, 31349–31358; 22810237 10.1074/jbc.M112.386706 PMC3438964

[8f] S. A. Polizzi , R. M. Walsh, Jr. , W. B. Peeples , J.-M. Lim , L. Wells , Z. A. Wood , Biochemistry 2012, 51, 8844–8855. 23072385 10.1021/bi301135b PMC4932848

[9a] T. Eixelsberger , B. Nidetzky , Adv. Synth. Catal. 2014, 356, 3575–3584; 26190959 10.1002/adsc.201400766 PMC4498474

[9b] P. Wildberger , L. Brecker , B. Nidetzky , Carbohydr. Res. 2012, 356, 224–232. 22591555 10.1016/j.carres.2012.04.003

[10] K. L. Kavanagh , H. Jörnvall , B. Persson , U. Oppermann , Cell. Mol. Life Sci. 2008, 65, 3895–3906. 19011750 10.1007/s00018-008-8588-y PMC2792337

[11a] W. W. Cleland in Isotope Effects in Chemistry and Biology (Eds.: A. Kohen, H.-H. Limbach), CRC Press, Boca Raton, 2006, pp. 915–930;

[11b] D. W. Parkin in Enzyme Mechanism from Isotope Effects (Ed.: P. F. Cook), CRC Press, Boca Raton, 1991, pp. 269–290;

[11c] A. Hengge in Isotope Effects in Chemistry and Biology (Eds.: A. Kohen, H.-H. Limbach), CRC Press, Boca Raton, 2006, pp. 955–974.

[12a] J. W. Munos , X. Pu , S. O. Mansoorabadi , H. J. Kim , H.-w. Liu , J. Am. Chem. Soc. 2009, 131, 2048–2049; 19159292 10.1021/ja807987h PMC2650392

[12b] J. F. Biellmann , E. L. O'Connell , I. A. Rose , J. Am. Chem. Soc. 1969, 91, 6484–6488; 5345032 10.1021/ja01051a053

[12c] U. Wong , R. J. Cox , Angew. Chem. Int. Ed. 2007, 46, 4926–4929; 10.1002/anie.200700647 17516600

[12d] L. L. Lee , M. V. Vu , W. W. Cleland , Biochemistry 2000, 39, 4808–4820. 10769138 10.1021/bi992894+

[13] The possibility of an early release of intermediate **4** was noted in some studies of UAXS (Refs. [7a,b]). Likewise, partial release of **4** was reported from reactions of UXS (Ref. [7f]). However, intermediate release appears to be adventitious rather than mechanistically diagnostic. In the reactions performed herein, intermediate **4** was not detectable in solution.

[14a] H.-P. Wahl , H. Grisebach , Biochim. Biophys. Acta Enzymol. 1979, 568, 243–252; 10.1016/0005-2744(79)90291-2 109125

[14b] H. Grisebach , R. Schmid , Angew. Chem. Int. Ed. Engl. 1972, 11, 159–248; 4626331 10.1002/anie.197201591

