# Supplementary material for: Isotope Probing of the UDP‐Apiose/UDP‐Xylose Synthase Reaction: Evidence of a Mechanism via a Coupled Oxidation and Aldol Cleavage
Source: Angew Chem Int Ed Engl. 2017 Jan 19;56(9):2503–7. doi: 10.1002/anie.201609288 (PMC5324594; doi:10.1002/anie.201609288)
Supplement: Supplementary file 1 — Supplementary [file ANIE-56-2503-s001.pdf]

Angewandte Chemie
Eine Zeitschrift der Gesellschaft Deutscher Chemiker

Supporting Information

Isotope Probing of the UDP-Apiose/UDP-Xylose Synthase Reaction: Evidence of a Mechanism via a Coupled Oxidation and Aldol Cleavage

*Thomas Eixelsberger, Doroteja Horvat, Alexander Gutmann, Hansjörg Weber, and Bernd Nidetzky**

anie_201609288_sm_miscellaneous_information.pdf

Table of Contents

Supporting methods and experimental details 3

Supporting tables 9

Supporting schemes 11

Supporting figures 13

Supporting methods and experimental details

Chemicals, enzymes and strains

Isotope-labelled D-glucose was from Omicron Biochemicals (South Bend, Indiana, USA) and had purities of 99% (^{13}C-labeled) or 98% (^{2}H-labeled). UTP (> 99% purity) was from Carbosynth (Compton, UK). UDP-α-D-glucuronic acid (> 98% purity), 1,5-anhydro-2-deoxy-D-*arabino*-hex-1-enitol, 9,10-phenanthrenequinone (> 99% purity) and bovine serum albumin (> 98% purity) were from Sigma-Aldrich (Vienna, Austria). NAD^+ (> 98% purity) was from Roth (Karlsruhe, Germany). Deuterium oxide for NMR measurements (99.96% ^{2}H) was from Euriso-Top (Saint-Aubin Cedex, France). All other chemicals were either from Sigma-Aldrich or Roth and were of the highest purity available.

Bovine liver catalase (3809 U mg^{-1}), *S. cerevisiae* hexokinase (25 U mg^{-1}), rabbit muscle phosphoglucomutase (≥ 100 U mg^{-1}) and *S. cerevisiae* inorganic pyrophosphatase (≥ 500 U mg^{-1}) were purchased from Sigma-Aldrich. Calf intestine alkaline phosphatase was from New England Biolabs (Ipswich, MA, USA). Expression and purification of the recombinant human UDP-glucose dehydrogenase (hUGDH), *Candida tenuis* xylose reductase, *Cellulomonas uda* cellobiose phosphorylase and human UDP-xylose synthase (UXS) are described elsewhere.[1-4] Gene BLLJ_1074 coding for UTP-glucose-1-phosphate uridylyltransferase from *Bifidobacterium longum* subsp. *longum* JCM 1217 (UniProt: E8MIY8), cloned into a pET-30a vector using *Nde*I and *Xho*I restriction sites for expression with a *C*-terminally fused His_6 tag, was kindly provided by Dr. Motomitsu Kitaoka (National Agriculture and Food Research Organization, Japan). The complete gene was sequenced before creating an expression strain by transformation of electrocompetent *E. coli* BL21 Gold (DE3) cells.

The sequence of gene AXS1 (GenBank: NM_128345) from *Arabidopsis thaliana*, coding for UAXS, was codon-optimized for expression in *E. coli* and a synthetic gene was ordered from GenScript (Piscataway, New Jersey, USA). The gene was cloned in a pET-26b(+) vector by the supplier using *Nde*I and *Xho*I restriction sites, fusing it to a *C*-terminal His_6 tag. After transformation of electrocompetent *E. coli* BL21 Gold (DE3) cells, the construct was verified by sequencing analysis (LGC Genomics, Berlin, Germany). For long-term storage, cells from a liquid culture (see below) were frozen with 40% (v/v) glycerol and stored at -70 °C.

Expression and purification of UAXS

Recombinant production of the enzyme was done according to established protocols.[4] A pre-culture containing 50 mL LB medium (50 mg L^{-1} kanamycin) in a 300 mL baffled flask was inoculated and grown overnight at 37 °C and 130 rpm. Main cultures contained 250 mL LB medium with kanamycin in 1 L baffled flasks and were inoculated to an OD_{600} of 0.01. The cultures were incubated at 37 °C and 130 rpm until the OD_{600} reached a value of 0.6-0.8, when 250 μM IPTG were added and the temperature

was decreased to 18 °C. Afterwards, expression of UAXS was performed overnight in a Certomat BS-1 incubator (Sartorius Stedim).

Cells were harvested by centrifugation at 5000 rpm and 4 °C using a Sorvall Evolution RC centrifuge (Thermo Fisher Scientific, Waltham, Massachusetts, USA) equipped with a F10-S rotor and the pellet was stored at -20 °C. For purification, the cells were disrupted by a French Pressure Cell Press (American Instrument Company, Silver Spring, Maryland, USA) and solid parts were removed by centrifugation (16000 g, 4 °C, 60 min) on an Eppendorf 5415 R centrifuge (Eppendorf). The supernatant was filtered through a 1.2 µm Minisart filter (Sartorius Stedim) and loaded on a self-packed Cu^{2+}-loaded IMAC sepharose column (GE Healthcare) using a BioLogic DuoFlow liquid chromatograph (Bio-Rad, Vienna, Austria). Elution was done using 50 mM Tris/HCl pH 7.5 buffers containing 5% (v/v) glycerol (buffer A and B) and 400 mM imidazole (buffer B). UAXS started eluting at 120 mM imidazole. All enzyme-containing fractions were pooled and the buffer was exchanged to 50 mM Tris/HCl pH 7.5 containing 5% (v/v) glycerol and 1 mM dithiothreitol using Amicon Ultra-15 centrifugal concentrators (Millipore, Vienna, Austria). Enzyme preparations were stored at -70 °C. The purity of the enzyme was checked with SDS-PAGE (NuPAGE 4-12% Bis-Tris-Gel, Life Technologies, Vienna, Austria) and Silver Staining (Figure S1). UAXS concentrations were determined by UV spectroscopy (λ = 280 nm) on a DeNovix DS-11+ microvolume spectrophotometer (DeNovix) using a molar extinction coefficient ϵ of 48360 $M^{-1}cm^{-1}$ and a molecular mass of 44703 Da (calculated by the ExPASy ProtParam web service).

Synthesis of UDP-α-D-glucuronic acid (3)

The synthesis is shown schematically in Scheme S1. The method for enzymatic synthesis of UDP-α-D-glucose was adapted from literature.[5,6] The reaction mixture (3 mL) contained 15 mM (isotope-labeled) monosaccharide (8.11 mg, 0.045 mmol), 40-50 mM uridine 5'-triphosphate (UTP; 58.1-72.6 mg, 0.120-0.150 mmol), 5 mM $MgCl_2$ (1.43 mg, 0.015 mmol), 10 µM α-D-glucose 1,6-bisphosphate and 0.13% (w/v) bovine serum albumin (BSA; 3.9 mg) dissolved in 50 mM Tris/HCl buffer (pH 7.5). 12 U mL^{-1} hexokinase (36 U), 6 U mL^{-1} phosphoglucomutase (18 U), 1.2 U mL^{-1} UTP-glucose-1-phosphate uridylyltransferase (UGPase; 3.6 U) and 1.2 U mL^{-1} inorganic pyrophosphatase (PPase; 3.6 U) were added and the reaction was incubated at 30 °C for 24 hrs (Figure S2). Upon completion, the mixture was split in three 1-mL-batches and synthesis of compound **3** was started by increasing temperature to 37 °C and adding 2 mM NAD^+ (1.33 mg, 0.002 mmol), 25 µM 9,10-phenanthrenequinone, 20 mM hydrogen peroxide (H_2O_2; 0.68 mg, 0.020 mmol), 2.4 U mL^{-1} xylose reductase (*Ct*XR; 2.4 U), 100 U mL^{-1} catalase (100 U) and 0.9 U mL^{-1} UDP-glucose dehydrogenase (hUGDH; 0.9 U) to each batch. The mixtures were incubated for 180 min and H_2O_2 was fed in regular intervals, as described previously (Figure S3).[7]

Prior to chromatographic purification of **3**, enzymes were removed by ultrafiltration using Vivaspin-6 centrifugal concentrators (Sartorius Stedim, Vienna, Austria). Purification of the produced nucleotide

sugars was done using an ÄKTA FPLC liquid chromatograph (GE Healthcare, Vienna, Austria) equipped with a 5 mL HiTrap Q HP (GE Healthcare) anion exchange column and a 2 mL sample loop. A step-wise gradient of 7.5 mL min^{-1} sodium chloride (buffer A: 20 mM and buffer B: 500 mM) in a 20 mM sodium acetate buffer (pH 4.2) was used for elution of bound compounds (Figure S4). The steps were as follows: 6 mL NaCl 0 mM, 36.5 mL NaCl 75 mM, 26 mL NaCl 125 mM, 15 mL NaCl 500 mM, 15 mL NaCl 0 mM. Prior to each run, the column was regenerated flushing it with at least three column volumes of buffer B and buffer A. UV absorption (λ = 254 nm) was used to detect the target compounds, which were collected. All product-containing fractions were pooled and concentrated on a Laborota 4000 rotary evaporator (Heidolph, Schwabach, Germany) at 45 °C and 20 mbar to a final volume of approximately 4 mL.

NaCl was removed from nucleotide sugar preparations using ÄKTA FPLC with a 2 mL sample loop and a Superdex Peptide 10/300 GL size exclusion column (GE Healthcare). Elution was performed with deionized water at a flow rate of 1 mL min^{-1}. The target compound was detected by UV absorption (λ = 254 nm, Figure S5). Product-containing fractions were collected, pooled and concentrated on the Laborota 4000 at 45 °C and 20 mbar to a final volume of approximately 5-10 mL. Residual H_2O was removed by lyophilization on a Christ Alpha 1-4 lyophilizer (B. Braun Biotech International, Melsungen, Germany), after which the sugar nucleotide product was obtained as white powder. NMR data are given in Figures S6 - S11.

Synthesis of UDP-2-deoxy-α-D-glucuronic acid (2-deoxy-3)

The synthesis is schematically shown in Scheme S2. 1,5-Anhydro-2-deoxy-D-*arabino*-hex-1-enitol was converted to 2-deoxy-α-D-glucose 1-phosphate by cellobiose phosphorylase (*Cu*CPase) using a modification of the method published by Wildberger *et al.*[3], and further on to UDP-2-deoxy-α-D-glucose by UGPase, as described for synthesis of substrate **3**. The reaction mixture (3 × 1 mL) contained 40 mM glycal substrate (17.5 mg, 0.120 mmol), 20 mM UTP (29.1 mg, 0.060 mmol), 5 mM $MgCl_2$ (1.43 mg, 0.015 mmol) and 0.13% (w/v) BSA (3.9 mg) dissolved in 31 mM potassium phosphate buffer (pH 7.0). After addition of 0.5 U mL^{-1} inorganic PPase (1.5 U), 84.5 U mL^{-1} UGPase (253.5 U) and 15 μM *Cu*CPase (0.045 μmol), the reaction was incubated at 30 °C for 70 hrs. For synthesis of substrate analogue 2-deoxy-**3**, the pH was set to 7.5 and temperature was increased to 37 °C. After addition of 2 mM NAD^+ (1.33 mg, 0.002 mmol), 25 μM 9,10-phenanthrenequinone, 20 mM H_2O_2 (0.68 mg, 0.020 mmol), 2.64 U mL^{-1} *Ct*XR (2.64 U), 100 U mL^{-1} catalase (100 U) and 1.8 U mL^{-1} hUGDH (1.8 U) to each batch, the mixtures were incubated for 5.5 hrs. H_2O_2 was fed in regular intervals as described previously.[7] A synthesis time course is shown in Figure S12.

Purification was done according to substrate **3**, however, a 1 mL SuperQ 650M column (Tosoh Bioscience GmbH, Stuttgart, Germany), a flow rate of 3 mL min^{-1} and the following steps were used: 16 mL

NaCl 0 mM, 92 mL NaCl 20 mM, 20 mL NaCl 150 mM, 17 mL NaCl 500 mM, 16 mL NaCl mM (Figure S13). NaCl was separated from compound 2-deoxy-**3** as described for substrate **3** (Figure S14). Water was always removed under N_2 flow due to the instability of 2-deoxy-**3**. NMR and HPLC data are shown in Figures S15 - S18.

UAXS enzymatic reactions and assays

Reactions were performed at 30 °C on a Thermomixer Comfort (Eppendorf, Hamburg, Germany) without agitation. The reaction volume was typically 500 µL. If not stated otherwise, 2.0 mM of substrate were used. The buffer was 50 mM potassium phosphate, pH 8.5. In the case that D_2O was used, the pD was set as pH meter reading plus 0.4. Purified UAXS (20 µM) was added to start the reaction and a sample was drawn immediately. Further samples were taken in regular intervals. For stopping the reaction, samples were heated to 99 °C for 5 min or mixed with acetonitrile (1:1 ratio). The samples were analyzed by HPLC as described below. Concentrations of substrate stock solutions were always confirmed by UV spectroscopy (λ = 262 nm) on a DeNovix DS-11+ microvolume spectrophotometer (DeNovix, Wilmington, Delaware, USA) using a molar extinction coefficient ε of 10 $mM^{-1}cm^{-1}$, as described in literature.[8]

For measuring the pH profile of the activity of UAXS, the phosphate buffer was used in the pH range 6.0 - 8.5, 50 mM sodium citrate was used between pH 5.0 and 6.0, and 50 mM Tris/HCl was used between pH 8.5 and 9.5. Reactions were performed identically as described above.

In the case of using UDP-2-deoxy-GlcA as the substrate of UAXS, alkaline phosphatase was added to the reaction (10 U mL^{-1}) to convert any UDP present into uridine and phosphate. The UDP originates from spontaneous degradation of the unstable substrate, and it inhibits the enzyme.

High performance liquid chromatography

Samples were analyzed on a Shimadzu Prominence HPLC system (Shimadzu, Korneuburg, Austria) equipped with a 5 µm Kinetex C18 analytical HPLC column (4.6 × 50 mm; Phenomenex, Aschaffenburg, Germany) and a UV detector (λ = 262 nm). Precipitated protein was removed from samples by centrifugation (16000 g, 4 °C, 5 min) on an Eppendorf 5415 R centrifuge (Eppendorf), and after proper dilution, samples were measured using an injection volume of 5 µL, a temperature of 35 °C and a flow rate of 2 mL min^{-1}. Isocratic elution was performed using 87.5% 40 mM tetra-*n*-butylammonium bromide in 20 mM potassium phosphate buffer (pH 5.9) and 12.5% acetonitrile. Analysis time was 3.5 min (without UTP) or 4.5 min (with UTP). Authentic standards were used for calibration.

KIEs from intermolecular competition experiments: reactions and sample preparation

Reaction conditions were the same as for standard enzymatic assays, except that approximately 1 mM unlabeled substrate **3** and approximately 1 mM 3-^2H or 4-^2H substrate **3** were mixed to yield the final

substrate concentration. After the reaction had proceeded to the desired level (10 - 70% conversion), enzymes were removed by ultrafiltration, as described for synthesis of substrate **3**. Purification of the remaining substrate or the reaction products was done using an ÄKTA FPLC liquid chromatograph (GE Healthcare) equipped with a 5 mL HiTrap Q HP anion exchange column (GE Healthcare) and a 2 mL sample loop (Figure S19). A step gradient of 7.5 mL min^{-1} ammonium formate buffer (buffer A: 20 mM and buffer B: 500 mM; pH 4.2) was used for elution of bound compounds (Figure S4). Collected 5-mL-fractions were lyophilized and subjected to desalting on a Superdex Peptide 10/300 GL column (GE Healthcare), as described above. After lyophilization and dissolution in D_2O, all fractions were analyzed by NMR-spectroscopy. The isolation procedure was also applied to unreacted substrate and shown not introduce an isotope effect.

NMR spectroscopy *(including in-situ analysis of enzymatic reactions)*[9]

A Varian (Agilent) INOVA 500-MHz NMR spectrometer (Agilent Technologies, Santa Clara, California, USA) and the VNMRJ 2.2D software were used for all measurements. ^{1}H NMR spectra (499.98 MHz) were measured on a 5 mm indirect detection PFG-probe, while a 5 mm dual direct detection probe with z-gradients was used for ^{13}C NMR spectra (125.71 MHz). NMR data were recorded from purified substances or reaction mixtures. In addition, they were also recorded from enzymatic in situ reactions performed at 30 °C in a total volume of 500 µL potassium phosphate buffer (50 mM; pD 8.5) in D_2O containing 2 mM of the respective substrate and 20 - 100 µM UAXS. ^{1}H NMR spectra were recorded with pre-saturation of the water signal by a shaped pulse in case of in situ experiments. Standard pre-saturation sequence was used: relaxation delay 2 s; 90° proton pulse; 2.048 s acquisition time; spectral width 8 kHz; number of points 32 k. ^{13}C NMR spectra during in situ experiments were recorded with the following pulse sequence: standard ^{13}C pulse sequence with 45° carbon pulse, relaxation delay 2 s, Waltz decoupling during acquisition, 2 s acquisition time. Up to 256 scans were accumulated in one measurement. Arrayed spectra were acquired with an array of pre-acquisition delay of 30 min or 60 min. HSQC spectra were measured with 128 scans per increment and adiabatic carbon 180° pulses. HETCOR spectra were recorded with 4 scans per increment and 256 increments. For KIE analysis, a standard proton experiment with relaxation delay of 25 s was used to record ^{1}H NMR spectra. ACD/NMR Processor Academic Edition 12.0 (Advanced Chemistry Development Inc.) was used for evaluation of spectra.

KIEs from intermolecular competition experiments: NMR analysis and KIE determination

Evaluation of the spectra for determination of the $^{1}H/^{2}H$ ratio was done using ACD/Processor Academic Edition NMR Processor Academic Edition 12.0 (Advanced Chemistry Development Inc., Toronto, Canada). Peaks were fitted with a Gauss+Lorentz function and optimized using a Levenberg-Marquadt algorithm, yielding peak areas. By comparison of the area of the isotopically replaced atom with the unaf-

fected atoms, the $^1H/^2H$ ratio was determined. The total concentration of substrate (UDP-GlcA) or product (UDP-Xyl; UDP-Api measured as UMP) was measured by HPLC and converted to the individual concentrations of ^1H and ^2H substrate using the ratio determined by NMR spectroscopy. KIEs were then calculated for each point applying the formula KIE = $\ln(1 - F_1)/\ln(1 - F_2)$ (measuring the residual substrate). F_1 and F_2 correspond to the fractional conversions of the ^1H and ^2H substrate, respectively. The KIE determined thus is on the catalytic efficiency (V_{max}/K_m).

KIEs from direct comparison of reaction rates

Enzymatic reactions were performed under the conditions described above (50 mM potassium phosphate buffer, pH 8.5) using unlabeled **3** or [4-^2H]-**3** as the substrate. The substrate concentration was saturating (≥ 1 mM) and varied at 4 concentrations in the range 1 - 3 mM. The conversion of substrate and the formation of products were measured by analyzing samples with HPLC at different times between 15 and 200 min. Initial reaction rates (*V*) were calculated from the linear dependencies of substrate consumed and time used. The KIE was calculated as the ratio of the *V* with unlabeled and deuterium-labeled substrate. The effect of substrate deuteration on the ratio of products formed from **3** was also analyzed.

Transient formation of enzyme-bound NADH

The UAXS as isolated contains tightly bound NAD^+. Judging from absorbance at 340 nm in the purified protein, the enzyme does not contain NADH. External NAD^+ was not necessary for activity and addition of NAD^+ (up to 500 μM) did not affect the enzymatic reaction rate. The formation of enzyme-bound NADH was monitored spectrophotometrically at 340 nm (Beckmann Coulter DU 800, Brea, CA, USA) during conversion of **3** under the conditions of the enzymatic assay described above. Using 20 μM UAXS and assuming a molar extinction coefficient of 6.22 mM^{-1} cm^{-1} for NADH, reduction of just 5 - 10% of the enzyme present would have been easily detectable. Reactions were also performed under rapid mixing conditions with an Applied Photophysics (Leatherhead, U.K.) model SX.18 MV Stopped-Flow Spectrometer. Enzyme was used in a limiting concentration (≤ 25 μM). Enzyme solution was mixed in 1:1 ratio with substrate solution, yielding concentrations of 2 mM of **3**. The absorbance of enzyme-bound NADH was measured at 340 nm.

Supporting tables

Table S1: Kinetic properties of UAXS dependent on pH

pH value[a]	UAXS specific activity[b]	
	$mU\ mg^{-1}$	%
5.5	2.10	23.2
6.5	2.23	24.6
7.5	3.05	33.6
8.5	9.07	100
9.5	2.36	26.0

[a] Buffers used: 50 mM citrate (pH 5.5), 50 mM phosphate (pH 6.5-8.5), 50 mM Tris/HCl (pH 9.5)
[b] Measured under standard conditions (2 mM substrate, 20 μM enzyme)

Table S2: Primary kinetic isotope effects on substrate consumption resulting from 4-^2H in substrate **3** determined in intermolecular competition experiments.

Conversion of **3**	4-^{2}H/4-^{1}H ratio in residual substrate **3**	Conversion of the 4-^{1}H (F_1) and the 4-^{2}H (F_2) substrate **3**	KIE = $\ln(1 - F_1)/\ln(1 - F_2)$
-	0.861	-	-
0.22	1.107	0.316 / 0.120	2.97
0.32	1.172	0.421 / 0.213	2.28
0.68	2.102	0.810 / 0.536	2.16

The average value of the KIE is 2.47 ± 0.43.

Table S3: Product ratios obtained from enzymatic reactions with unlabeled or deuterium-labeled substrate **3** determined at approximately 50% conversion.

Substrate	$\frac{[\text{Product 1}]^a}{[\text{Product 7}]}$
Unlabeled	1.84
3-^2H	1.92
4-^2H	1.48

[a] Measured as UMP

Table S4: Secondary kinetic isotope effects on substrate consumption resulting from 3-^2H in substrate **3** determined in intermolecular competition experiments.

Conversion of **3**	3-^{2}H/3-^{1}H ratio in residual substrate **3**	Conversion of the 3-^{1}H (F_1) and the 3-^{2}H (F_2) substrate **3**	KIE= $\ln(1 - F_1)/\ln(1 - F_2)$
-	0.736	-	-
0.18	0.765	0.196 / 0.165	1.21
0.47	0.812	0.494 / 0.442	1.17
0.52	0.846	0.549 / 0.481	1.21

The average value of the KIE is 1.20 ± 0.03.

Table S5: Isotope ratios on product formation resulting from 3-^{2}H in substrate **3** determined in intermolecular competition experiments.

Conversion of **3**	3-^{2}H/3-^{1}H ratio in **8**	3-^{2}H/3-^{1}H ratio in **7**
0.52	~0.64	~0.62
1.0	~0.74	~0.74

The measurement at full conversion of **3** serves as a control. The initial isotope Ratio 3-^{2}H/3-^{1}H in substrate **3** was 0.736 (see Table S4).

Supporting schemes

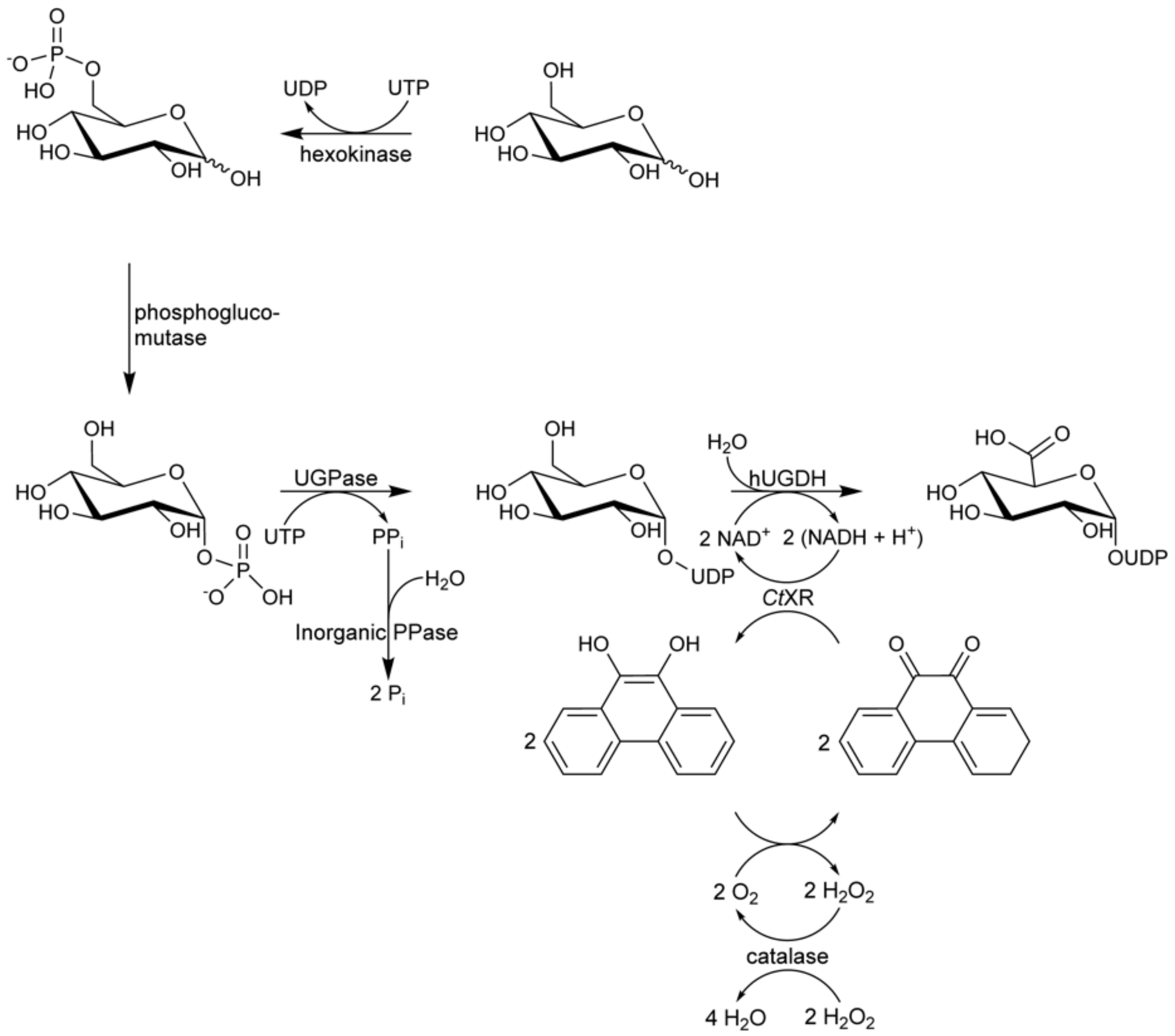

Scheme S1: The principle of enzymatic synthesis of substrate **3** and isotopically labeled analogues thereof is shown. UGPase, *Bifidobacterium longum* UTP-glucose-1-phosphate uridylyltransferase; inorganic PPase, pyrophosphatase; hUGDH, human UDP-glucose dehydrogenase; *Ct*XR, *Candida tenuis* xylose reductase.

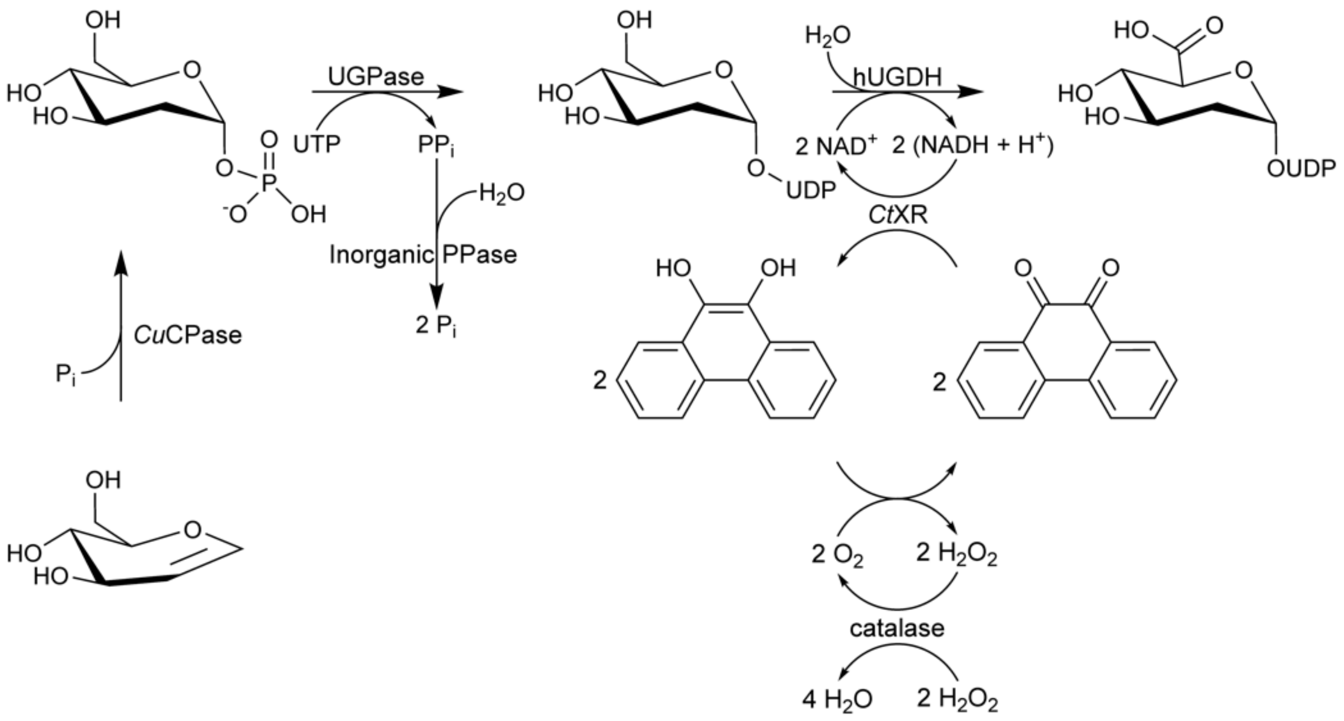

Scheme S2: The principle of enzymatic synthesis of substrate analogue 2-deoxy-**3** is shown. *Cu*CPase, *Cellulomonas uda* cellobiose phosphorylase; UGPase, *Bifidobacterium longum* UTP-glucose-1-phosphate uridylyltransferase; inorganic PPase, pyrophosphatase; hUGDH, human UDP-glucose dehydrogenase; *Ct*XR, *Candida tenuis* xylose reductase.

Supporting figures

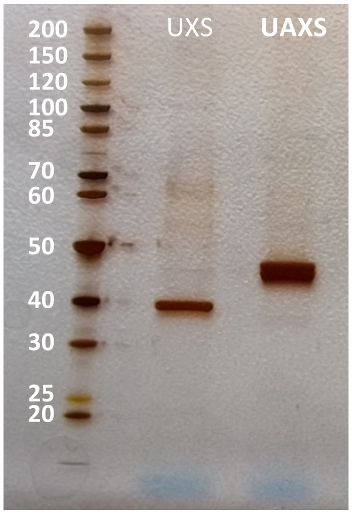

Figure S1. A SDS polyacrylamide gel of purified UAXS from *A. thaliana* (44.7 kDa) is shown. The gel also shows a molecular mass standard (masses in kDa) and the purified human UXS (38.6 kDa) that was also used in the current study.

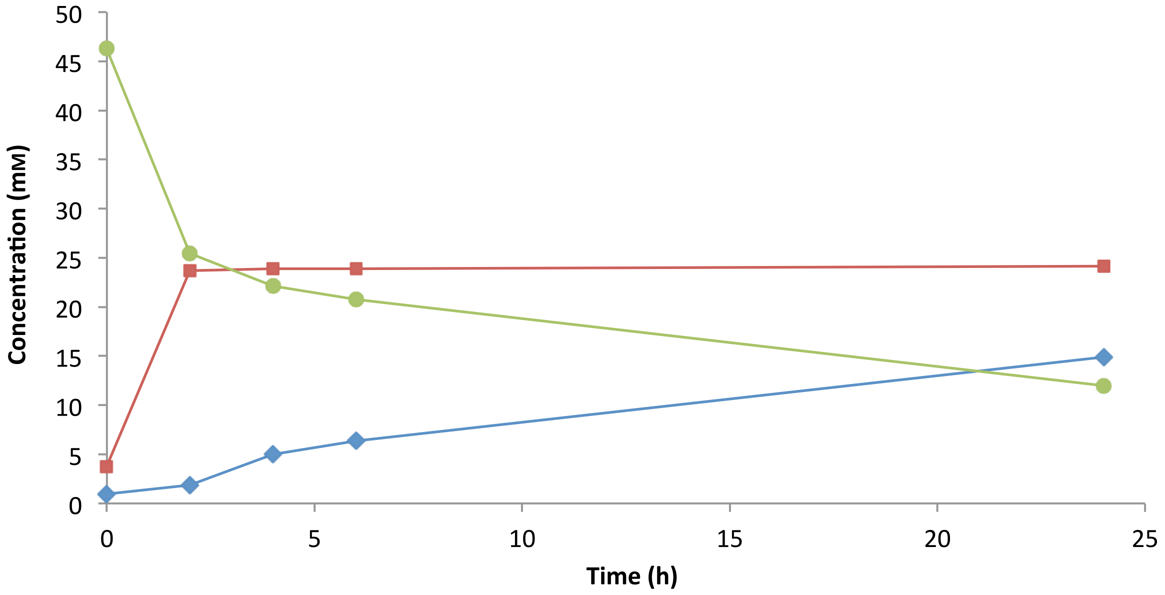

Figure S2. A representative time course of synthesis of UDP-α-D-glucose from 15 mM D-glucose via α-D-glucose 1-phosphate (blue: UDP-α-D-glucose, red: UDP, green: UTP) is shown.

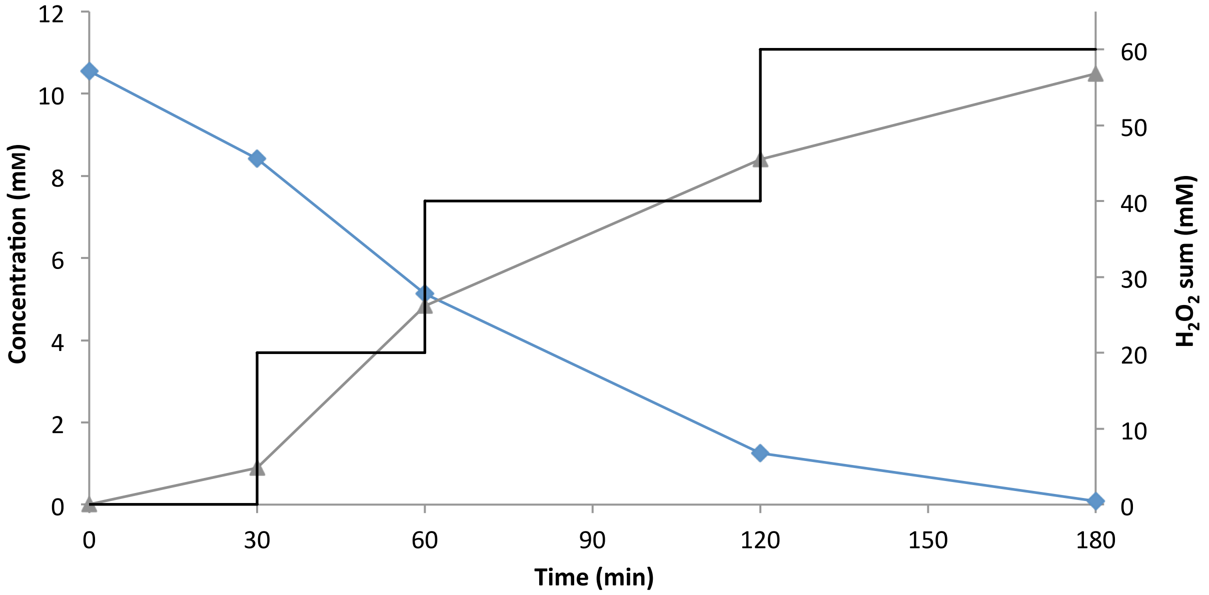

Figure S3. A representative time course of synthesis of substrate **3** from UDP-α-D-glucose is shown (blue: UDP-α-D-glucose, grey: substrate **3**, black: H_2O_2).

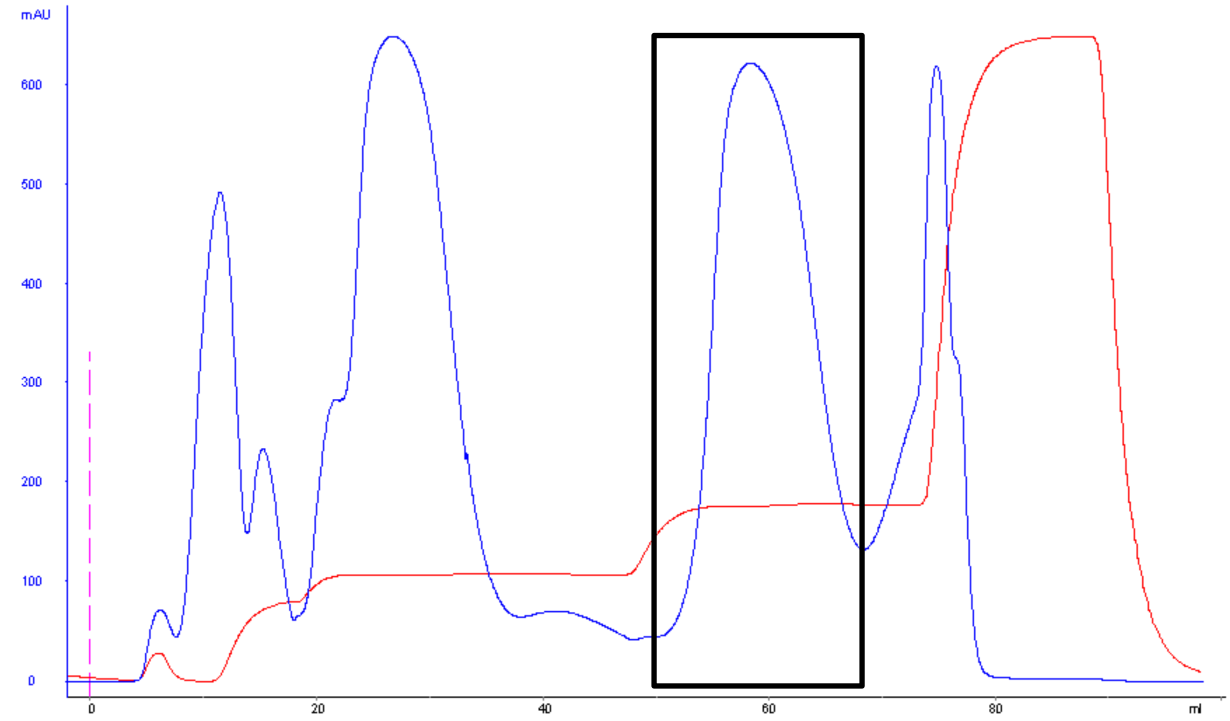

Figure S4. Purification of substrate **3** (indicated by black box) using anion exchange chromatography (blue: UV signal, red: conductivity signal) is shown.

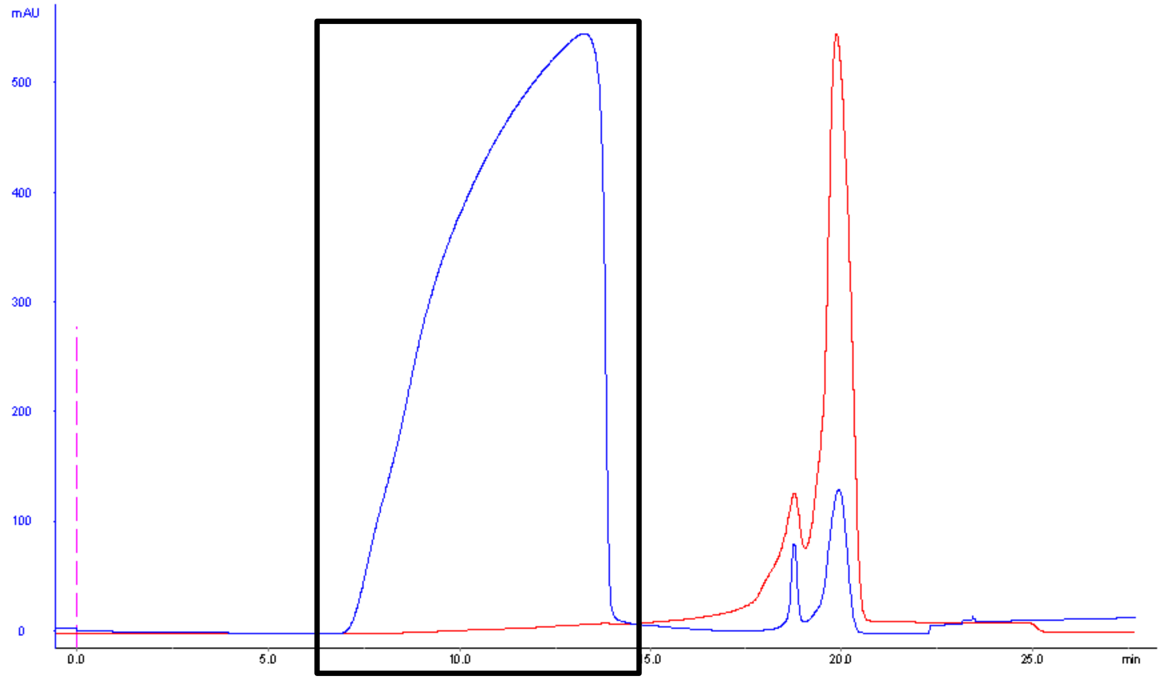

Figure S5. Removal of salt from substrate **3** by size exclusion chromatography is shown. Substrate **3** (indicated by black box) could be separated from NaCl in one step (blue: UV signal, red: conductivity signal).

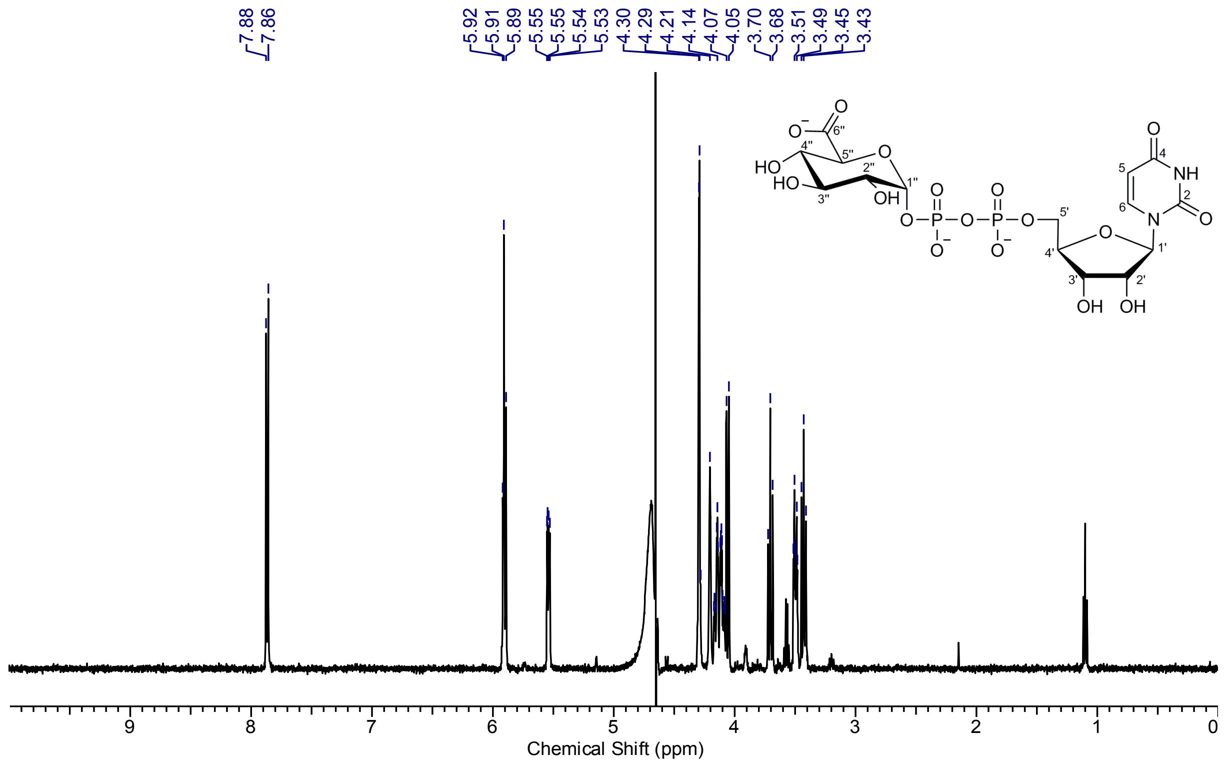

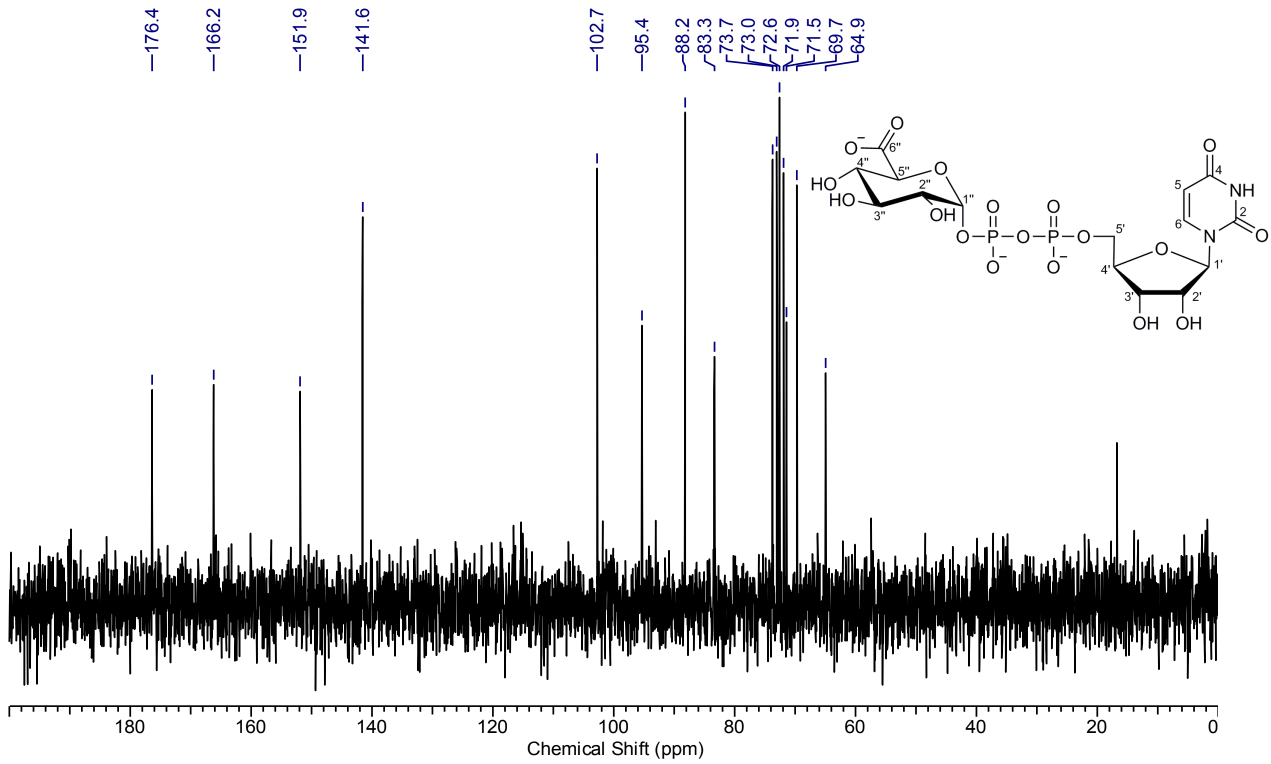

Figure S6. NMR analysis of the commercial substrate **3** (purchased from Sigma-Aldrich) is shown. ^{1}H NMR (500 MHz, D_2O) δ ppm 7.87 (d, *J*=8.30 Hz, 1 H, Ura H-6), 5.88 - 5.93 (m, 2 H, Ura H-5/Rib H-1'), 5.54 (dd, *J*=7.57, 3.66 Hz, 1 H, GlcA H-1''), 4.27 - 4.31 (m, 2 H, Rib H-2'/Rib H-3'), 4.21 (br. s., 1 H, Rib H-4'), 4.08 - 4.18 (m, 2 H, Rib H-5'), 4.06 (d, *J*=9.76 Hz, 1 H, GlcA H-5''), 3.71 (t, *J*=9.28 Hz, 1 H, GlcA H-3''), 3.50 (dt, *J*=9.76, 3.17 Hz, 1 H, GlcA H-2''), 3.43 (t, *J*=9.76 Hz, 1 H, GlcA H-4''); ^{13}C NMR (125 MHz, D_2O) δ ppm 176.4 (GlcA C-6''), 166.2 (Ura C-4), 151.9 (Ura C-2), 141.6 (Ura C-6), 102.7 (Ura C-5), 95.4 (GlcA C-1''), 88.2 (Rib C-1'), 83.3 (d, *J*=8.63 Hz, Rib C-4'), 73.7 (Rib C-3'), 73.0 (GlcA C-5''), 72.6 (GlcA C-3''), 71.9 (GlcA C-4''), 71.4 (d, *J*=7.67 Hz, GlcA C-2''), 69.7 (Rib C-2'), 64.9 (Rib C-5')

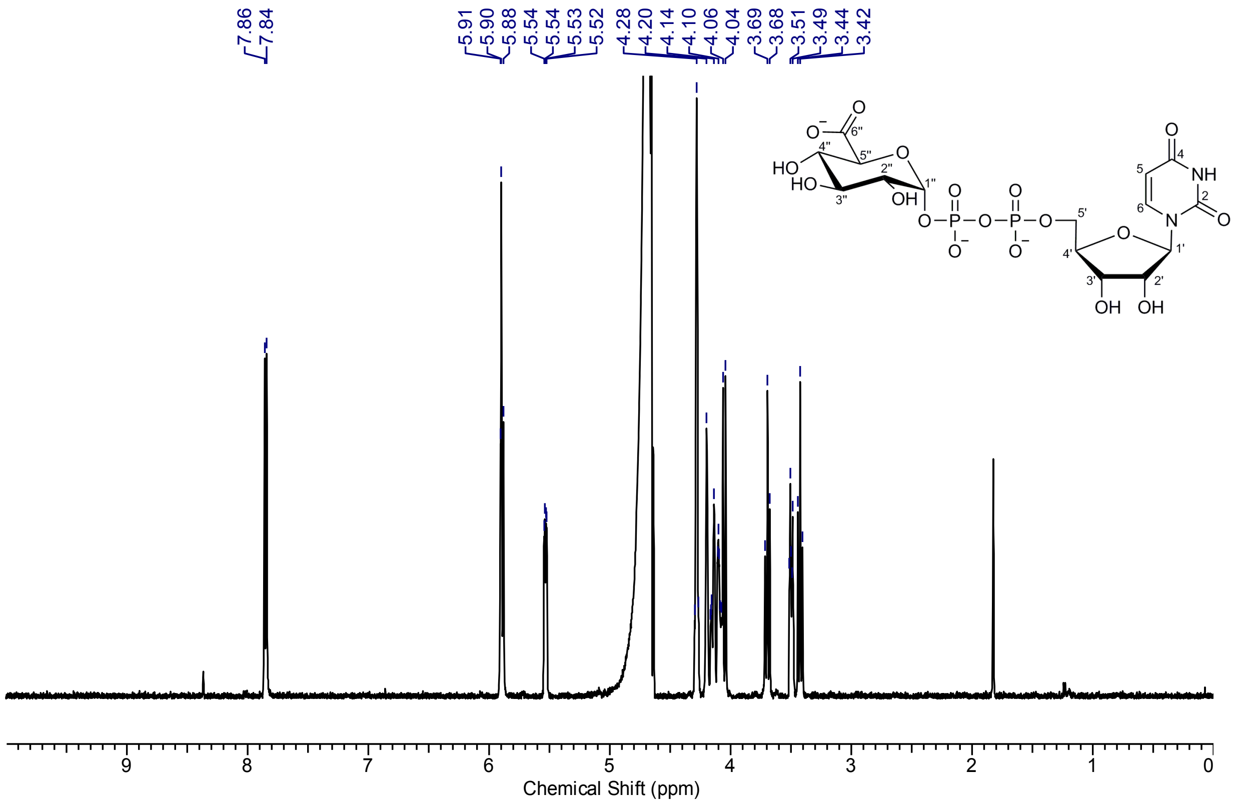

Figure S7. NMR analysis of the enzymatically synthesized unlabeled substrate **3** is shown. ^{1}H NMR (500 MHz, D_2O) δ ppm 7.85 (d, *J*=8.30 Hz, 1 H, Ura H-6), 5.87 - 5.92 (m, 2 H, Ura H-5/Rib H-1'), 5.53 (dd, *J*=7.32, 3.42 Hz, 1 H, GlcA H-1''), 4.26 - 4.30 (m, 2 H, Rib H-2'/Rib H-3'), 4.20 (br. s., 1 H, Rib H-4'), 4.07 - 4.17 (m, 2 H, Rib H-5'), 4.05 (d, *J*=10.25 Hz, 1 H, GlcA H-5''), 3.69 (t, *J*=9.28 Hz, 1 H, GlcA H-3''), 3.50 (dt, *J*=9.76, 2.93 Hz, 1 H, GlcA H-2''), 3.42 (t, *J*=9.76 Hz, 1 H, GlcA H-4'')

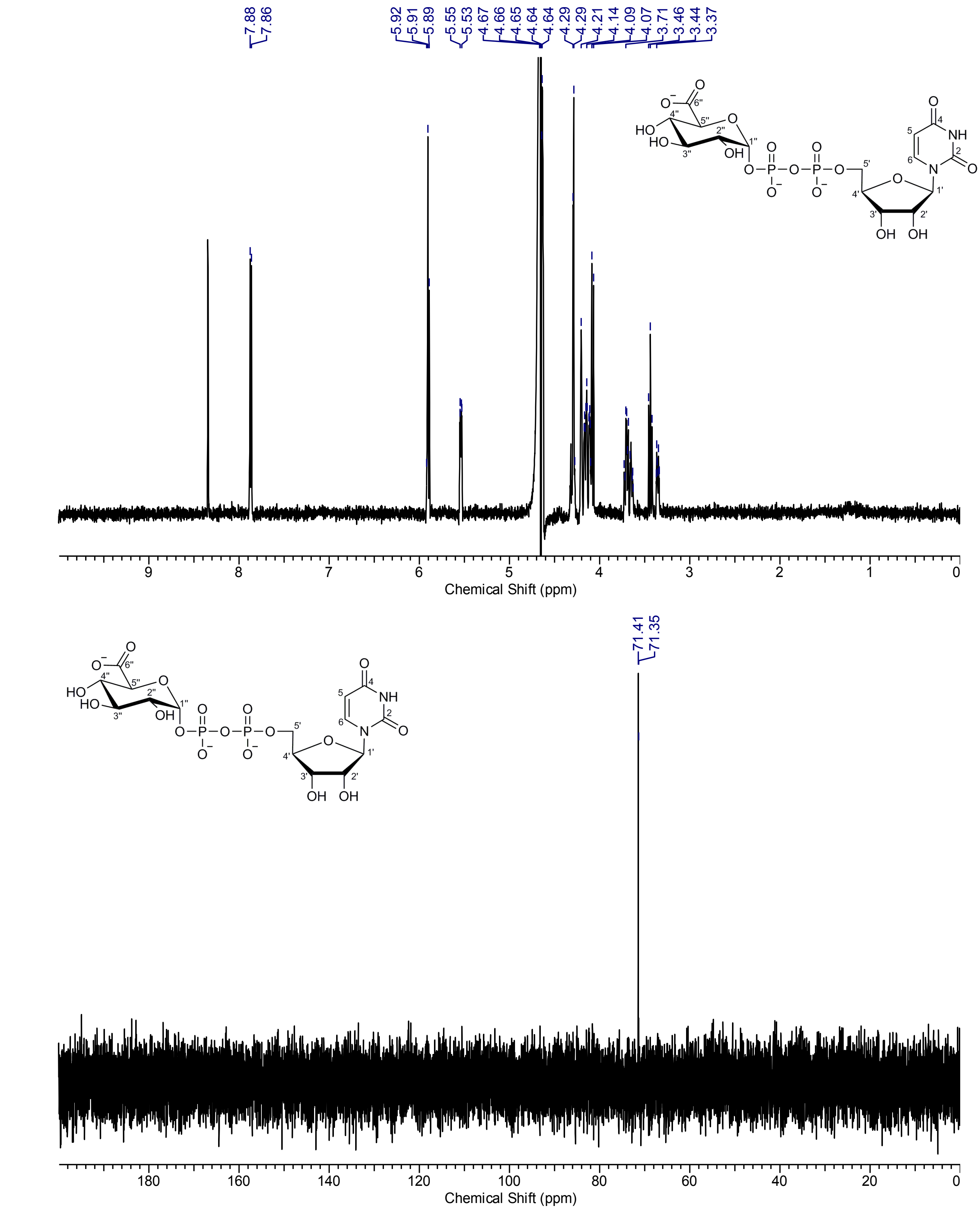

Figure S8. NMR analysis of the enzymatically synthesized 2-^{13}C substrate **3** is shown. ^{1}H NMR (500 MHz, D_2O) δ ppm 7.87 (d, *J*=8.30 Hz, 1 H, Ura H-6), 5.88 - 5.93 (m, 2 H, Ura H-5/Rib H-1'), 5.54 (dd, *J*=7.57, 2.20 Hz, 1 H, GlcA H-1''), 4.27 - 4.30 (m, 2 H, Rib H-2'/Rib H-3'), 4.21 (br. s., 1 H, Rib H-4'), 4.10 - 4.18 (m, 2 H, Rib H-5'), 4.08 (d, *J*=10.25 Hz, 1 H, GlcA H-5''), 3.67 - 3.74 (m, 1 H, GlcA H-3''), 3.64 (dt, *J*=9.76, 2.93 Hz, 0.5 H, GlcA H-2''), 3.44 (t, *J*=9.52 Hz, 1 H, GlcA H-4''), 3.36 (dt, *J*=9.76, 3.40 Hz, 0.5 H, GlcA H-2''); ^{13}C NMR (125 MHz, D_2O) δ ppm 71.4 (d, *J*=8.63 Hz, GlcA C-2'')

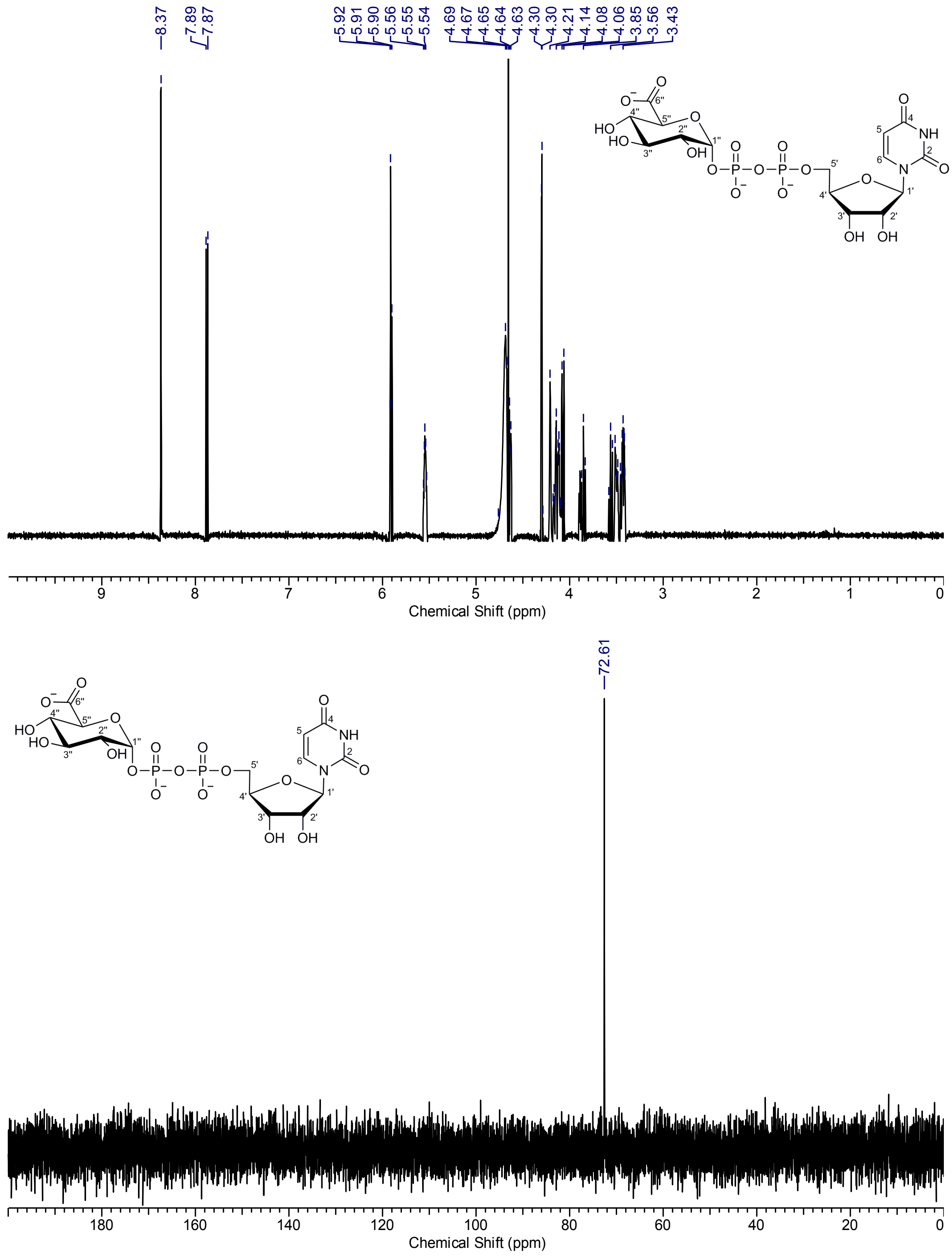

Figure S9. NMR analysis of the enzymatically synthesized 3-^{13}C substrate **3** is shown. ^{1}H NMR (500 MHz, D_2O) δ ppm 7.88 (d, *J*=8.30 Hz, 1 H, Ura H-6), 5.88 - 5.93 (m, 2 H, Ura H-5/Rib H-1'), 5.52 - 5.57 (m, 1 H, GlcA H-1''), 4.28 - 4.32 (m, 2 H, Rib H-2'/Rib H-3'), 4.21 (br. s., 1 H, Rib H-4'), 4.09 - 4.18 (m, 2 H, Rib H-5'), 4.07 (d, *J*=10.25 Hz, 1 H, GlcA H-5''), 3.86 (t, *J*=9.76 Hz, 0.5 H, GlcA H-3''), 3.56 (t, *J*=9.76 Hz, 0.5 H, GlcA H-3''), 3.50 (dt, *J*=13.18, 3.42 Hz, 1 H, GlcA H-2''), 3.40 - 3.47 (m, 1 H, GlcA H-4''); ^{13}C NMR (125 MHz, D_2O) δ ppm 72.6(GlcA C-3'')

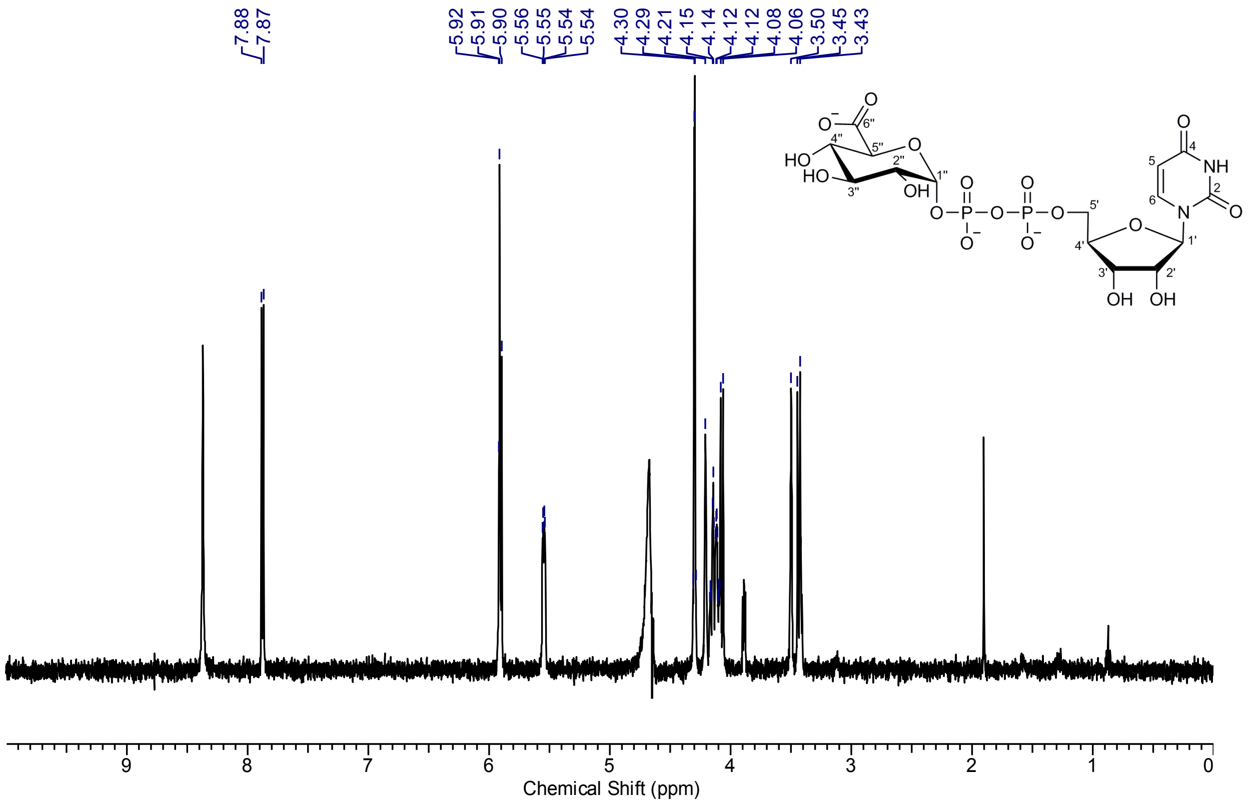

Figure S10. NMR analysis of the enzymatically synthesized 3-^{2}H substrate **3** is shown. ^{1}H NMR (500 MHz, D_2O) δ ppm 7.88 (d, *J*=8.30 Hz, 1 H, Ura H-6), 5.89 - 5.93 (m, 2 H, Ura H-5/Rib H-1'), 5.55 (dd, *J*=7.81, 2.93 Hz, 1 H, GlcA H-1''), 4.28 - 4.32 (m, 2 H, Rib H-2'/Rib H-3'), 4.21 (br. s., 1 H, Rib H-4'), 4.09 - 4.18 (m, 2 H, Rib H-5'), 4.07 (d, *J*=10.25 Hz, 1 H, GlcA H-5''), 3.50 (br. s., 1 H, GlcA H-2''), 3.44 (d, *J*=10.25 Hz, 1 H, GlcA H-4'')

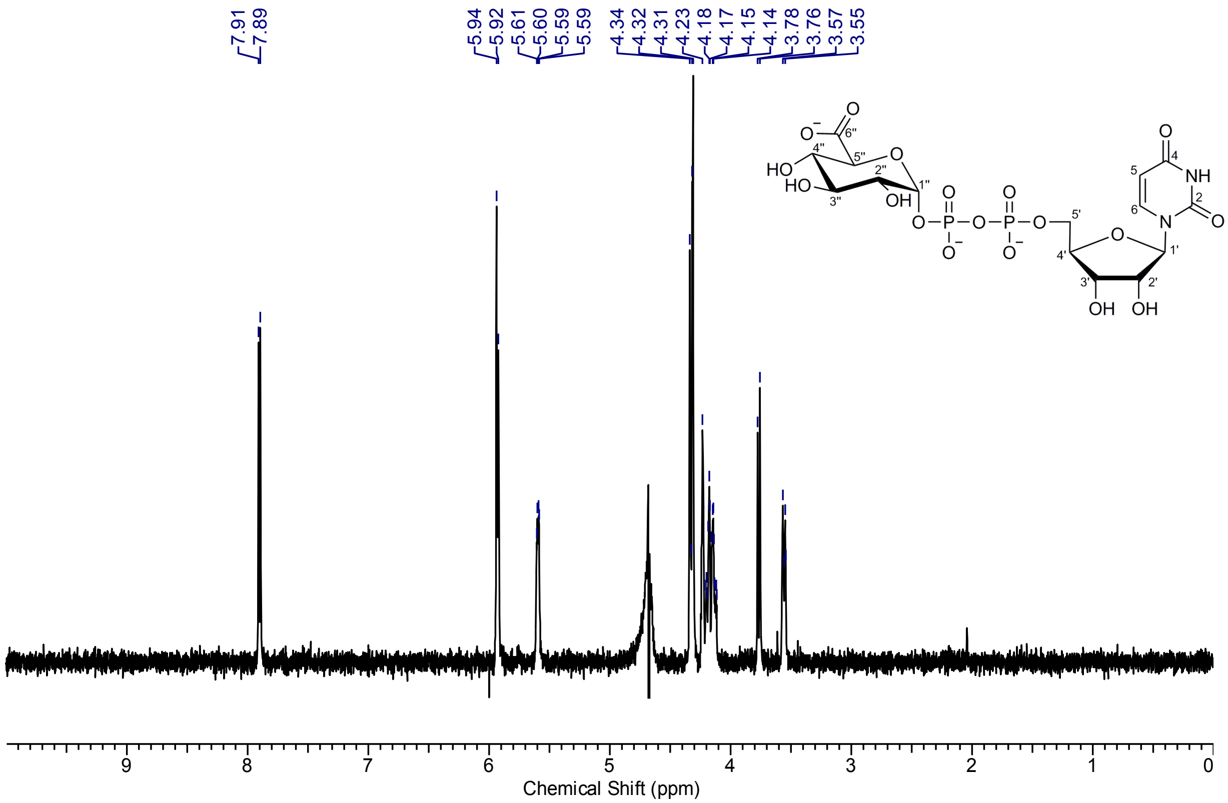

Figure S11. NMR analysis of the enzymatically synthesized 4-^2H substrate **3** is shown. ^{1}H NMR (500 MHz, D_2O) δ ppm 7.90 (d, *J*=8.30 Hz, 1 H, Ura H-6), 5.91 - 5.95 (m, 2 H, Ura H-5/Rib H-1'), 5.60 (dd, *J*=6.59, 3.17 Hz, 1 H, GlcA H-1''), 4.34 (s, 1 H, GlcA H-5''), 4.29 - 4.33 (m, 2 H, Rib H-2'/Rib H-3'), 4.23 (br. s., 1 H, Rib H-4'), 4.11 - 4.21 (m, 2 H, Rib H-5'), 3.77 (d, *J*=9.76 Hz, 1 H, GlcA H-3''), 3.55 (dt, *J*=9.28, 3.42 Hz, 1 H, GlcA H-2'')

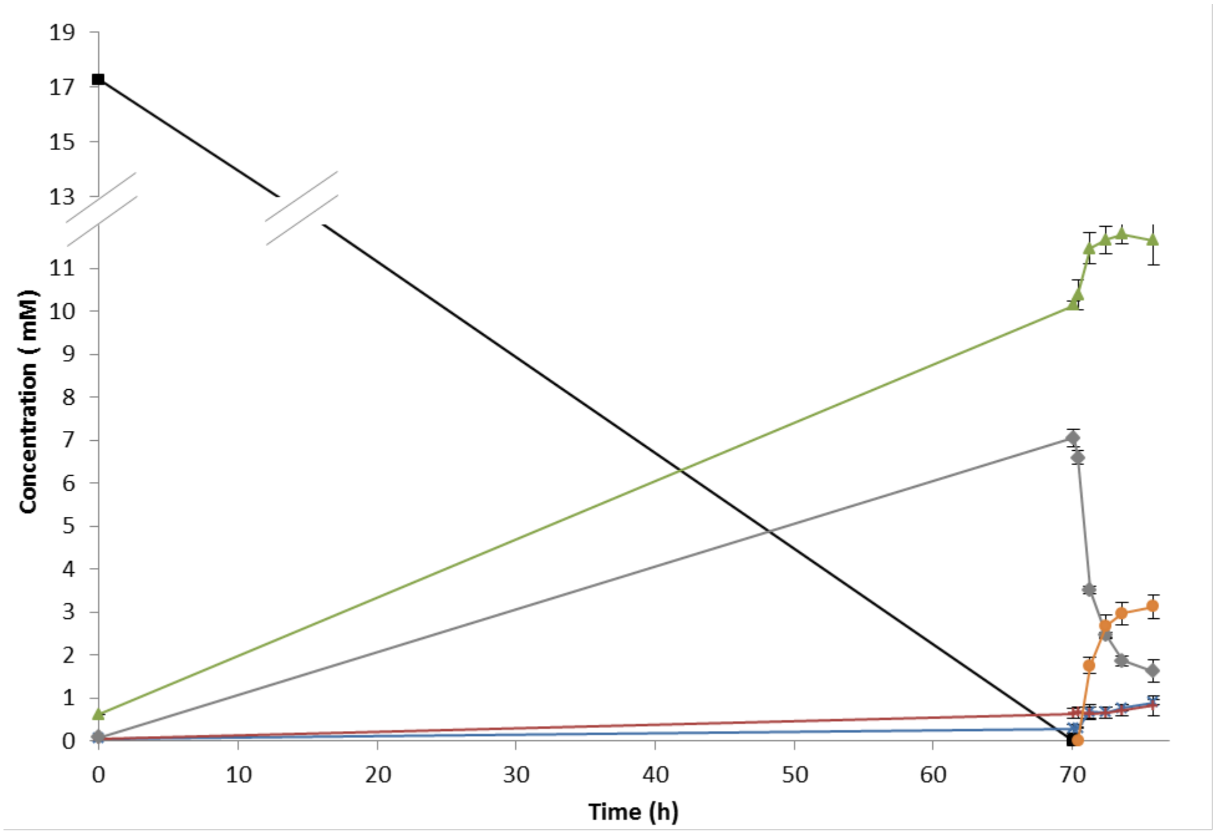

Figure S12. Time course of synthesis of 2-deoxy-**3** from UTP and 1,5-anhydro-2-deoxy-D-*arabino*-hex-1-enitol is shown (blue: uridine, red: UMP, green: UDP, black: UTP, grey: UDP-2-deoxy-α-D-glucose, orange: 2-deoxy-**3**).

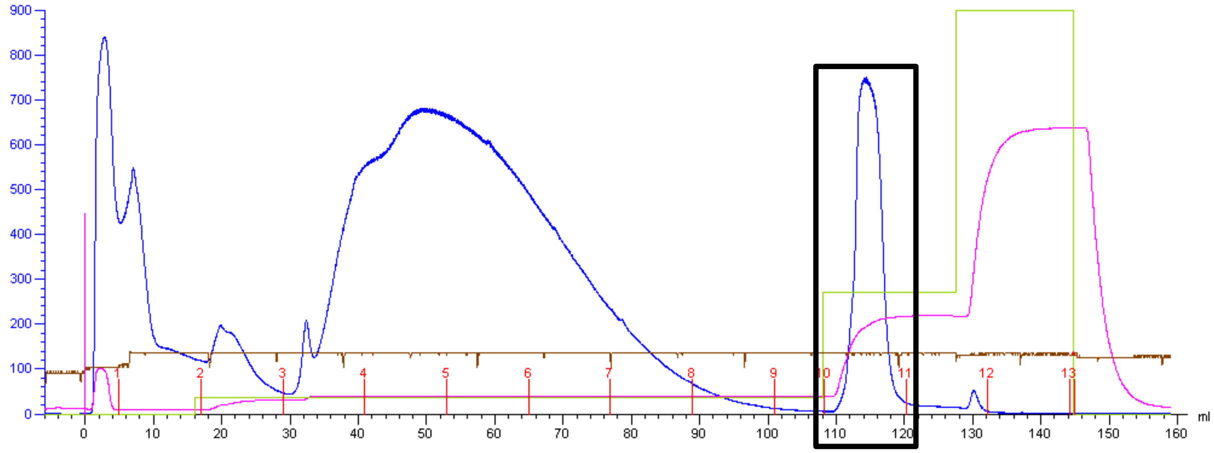

Figure S13. Purification of 2-deoxy-**3** (indicated by black box) using anion exchange chromatography (blue: UV signal, pink: conductivity signal, green: concentration buffer B) is shown.

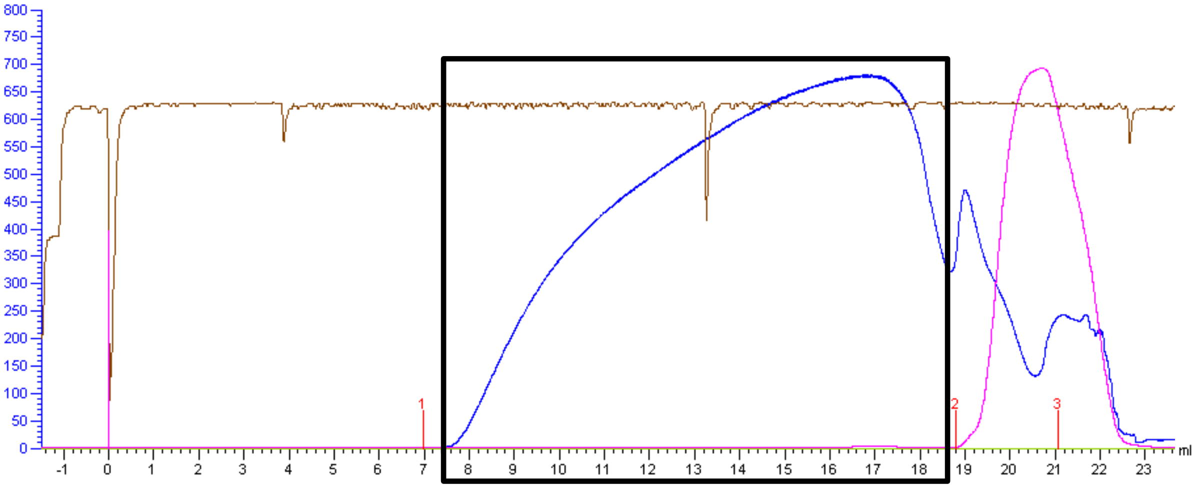

Figure S14. Removal of NaCl from 2-deoxy-**3** (indicated by black box) by size exclusion chromatography (blue: UV signal, pink: conductivity signal) is shown.

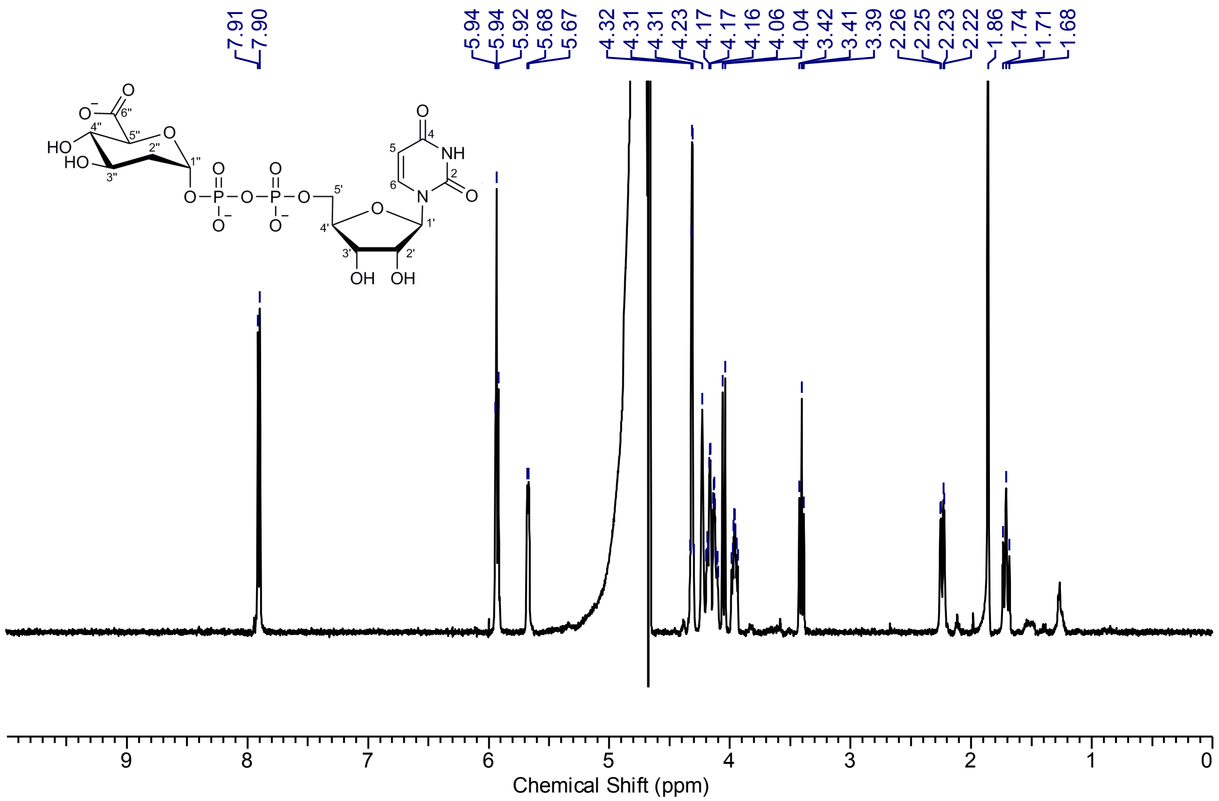

Figure S15. NMR analysis of the enzymatically synthesized 2-deoxy-**3** is shown. The singlet at 1.86 ppm is residual sodium acetate from purification. ^{1}H NMR (500 MHz, D_2O) δ ppm 7.91 (d, *J*=8.30 Hz, 1 H, Ura H-6), 5.90 - 5.97 (m, 2 H, Ura H-5/Rib H-1'), 5.67 (d, *J*=6.35 Hz, 1 H, Deoxy-GlcA H-1''), 4.29 - 4.35 (m, 2 H, Rib H-2'/Rib H-3'), 4.23 (br. s., 1 H, Rib H-4'), 4.09 - 4.21 (m, 2 H, Rib H-5'), 4.05 (d, *J*=9.76 Hz, 1 H, Deoxy-GlcA H-5''), 3.96 (ddd, *J*=11.35, 9.40, 5.13 Hz, 1 H, Deoxy-GlcA H-3''), 3.41 (t, *J*=9.52 Hz, 1 H, Deoxy-GlcA H-4''), 2.24 (dd, *J*=12.94, 4.64 Hz, 1 H, Deoxy-GlcA H-2''), 1.71 (t, *J*=12.69 Hz, 1 H, Deoxy-GlcA H-2'')

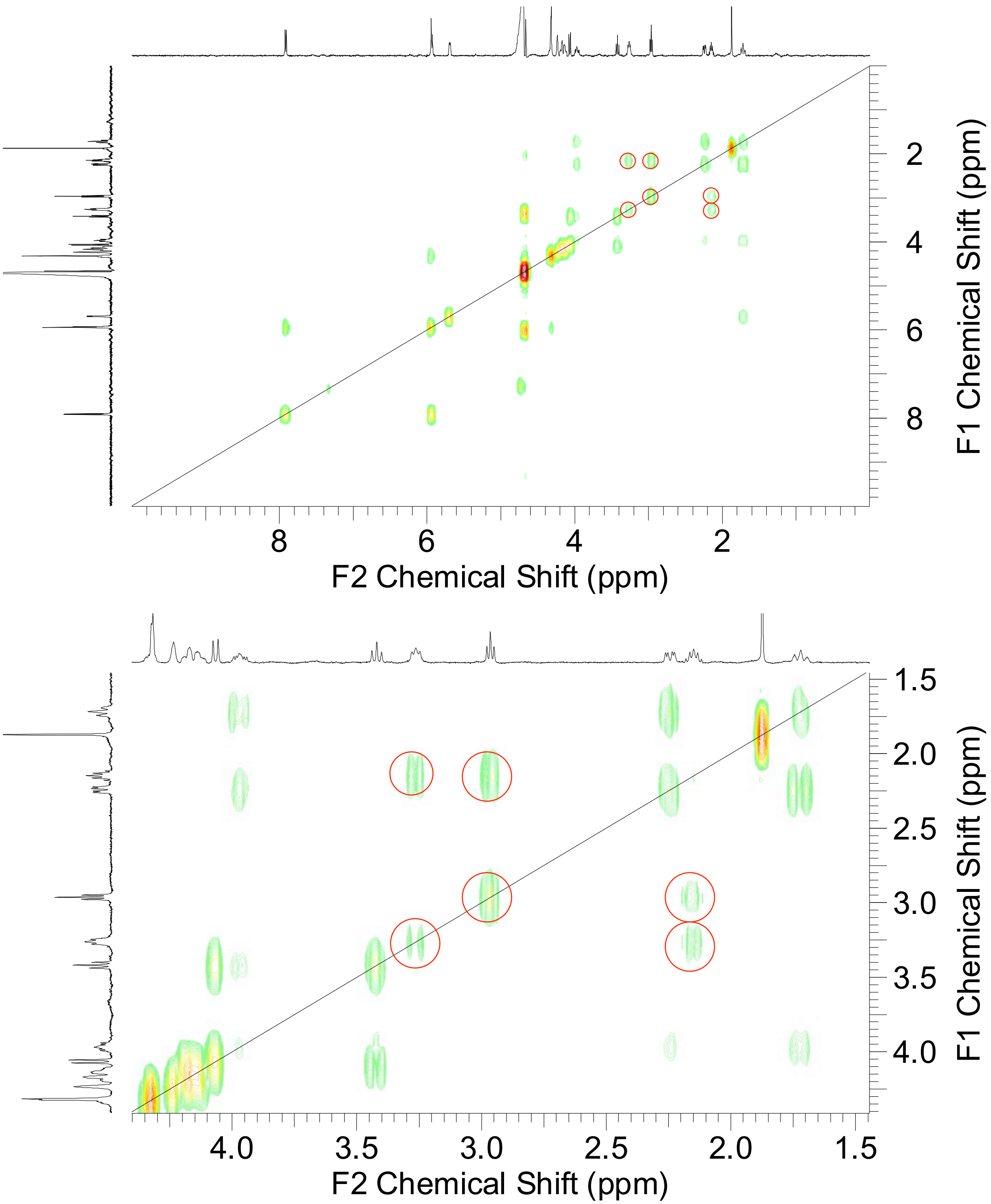

Figure S16. Correlation spectroscopy (COSY) spectrum of the synthesized 2-deoxy-**3** is shown. Signals from the buffer solution are indicated with red circles. Cross peaks (F2/F1) ppm 5.94/7.91 (Ura H-5/Ura H-6), 4.32/5.94 (Rib H-2'/Rib H-1'), 3.42/4.10 (Deoxy-GlcA H-4''/Deoxy-GlcA H-5''), 3.42/3.96 (Deoxy-GlcA H-4''/Deoxy-GlcA H-3''), 2.24/3.96 (Deoxy-GlcA H-2''/Deoxy-GlcA H-3''), 1.72/5.69 (Deoxy-GlcA H-2''/Deoxy-GlcA H-1''), 1.72/3.96 (Deoxy-GlcA H-2''/Deoxy-GlcA H-3''), 1.72/2.25 (Deoxy-GlcA H-2''/Deoxy-GlcA H-2''). Couplings between Rib H-2'/Rib H-3', Rib H-3'/Rib H-4' and Rib H-4'/Rib H-5' could not be resolved under the applied conditions.

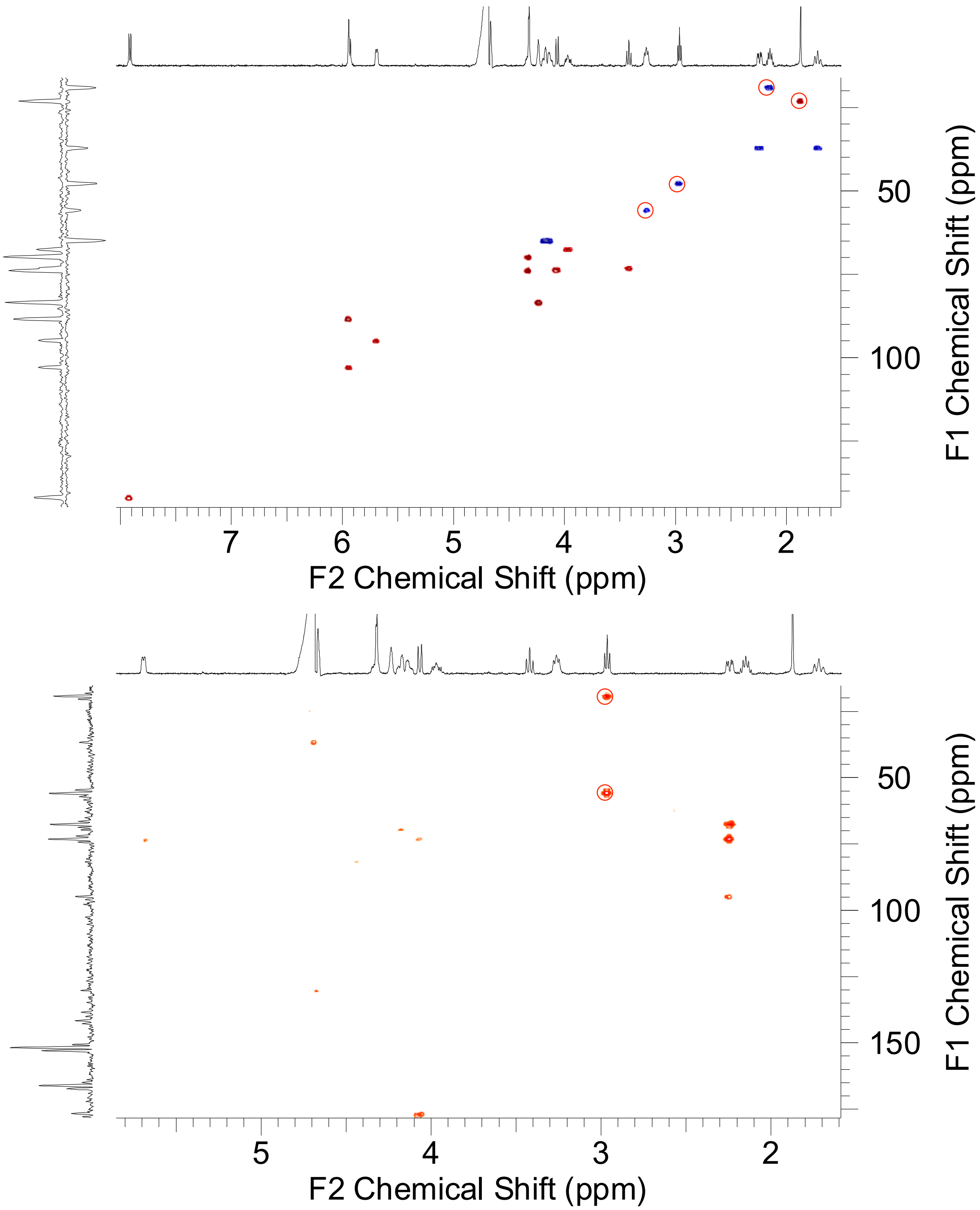

Figure S17. HSQC (top) and heteronuclear multiple-bond correlation (HMBC; down) spectra of the enzymatically synthesized 2-deoxy-**3** are shown (F1 axis = ^{13}C, F2 axis = ^{1}H). Signals from the buffer solution are indicated with red circles. HSQC (F2/F1) ppm 7.92/142.0 (Ura H/C-6), 5.94/103.1 (Ura H/C-5), 5.95/88.4 (Rib H/C-1'), 5.70/94.9 (Deoxy-GlcA H/C-1''), 4.33/73.9 (Rib H/C-3'), 4.33/70.0 (Rib H/C-2'), 4.24/83.5 (Rib H/C-4'), 4.17/65.0 (Rib H/C-5'), 4.07/73.9 (Deoxy-GlcA H/C-5''), 3.96/67.6 (Deoxy-GlcA H/C-3''), 3.42/73.3 (Deoxy-GlcA H/C-4''), 2.25/37.1 (Deoxy-GlcA H/C-2''), 1.72/37.2 (Deoxy-GlcA H/C-2'')

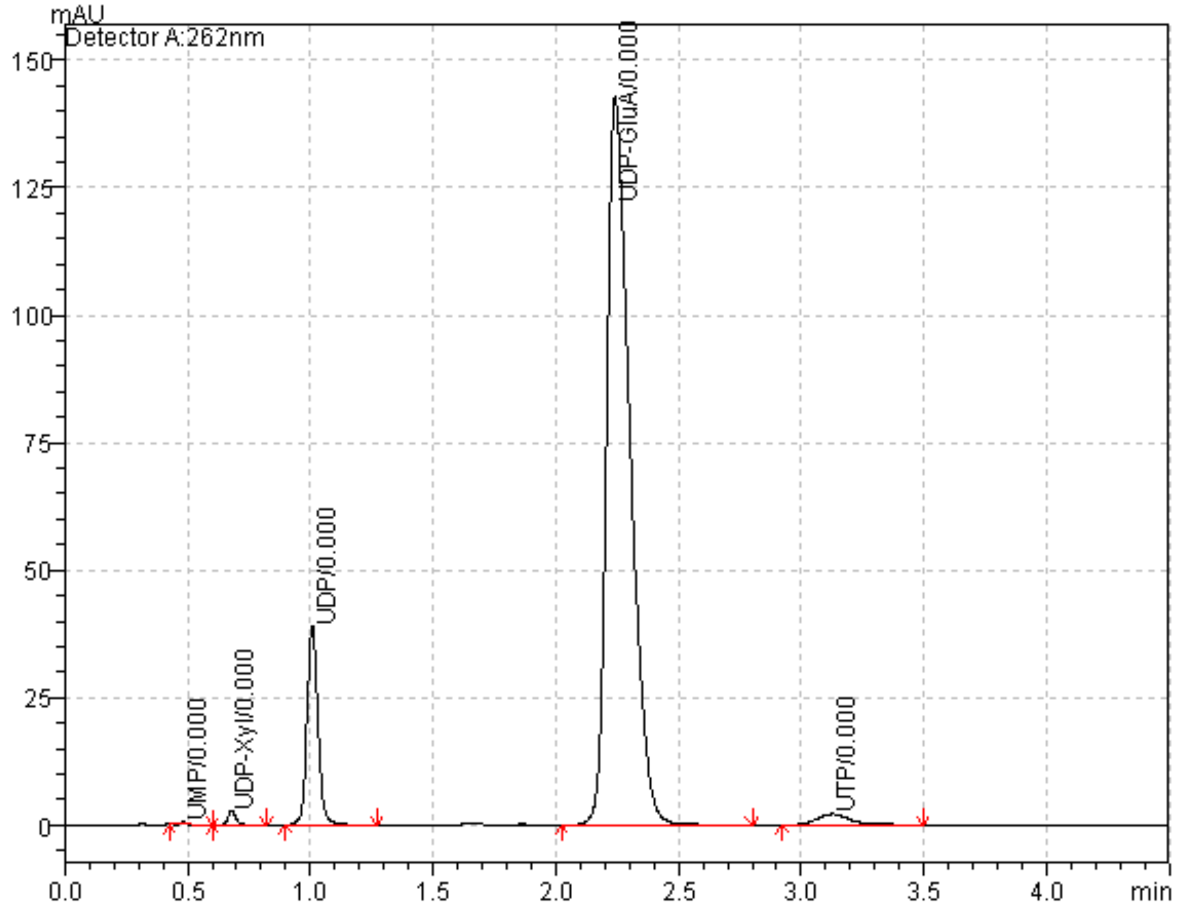

Figure S18. HPLC trace (UV detection, λ = 262 nm) of the enzymatically synthesized 2-deoxy-**3** is shown. UDP (< 15%) originates from partial decomposition during solvent removal.

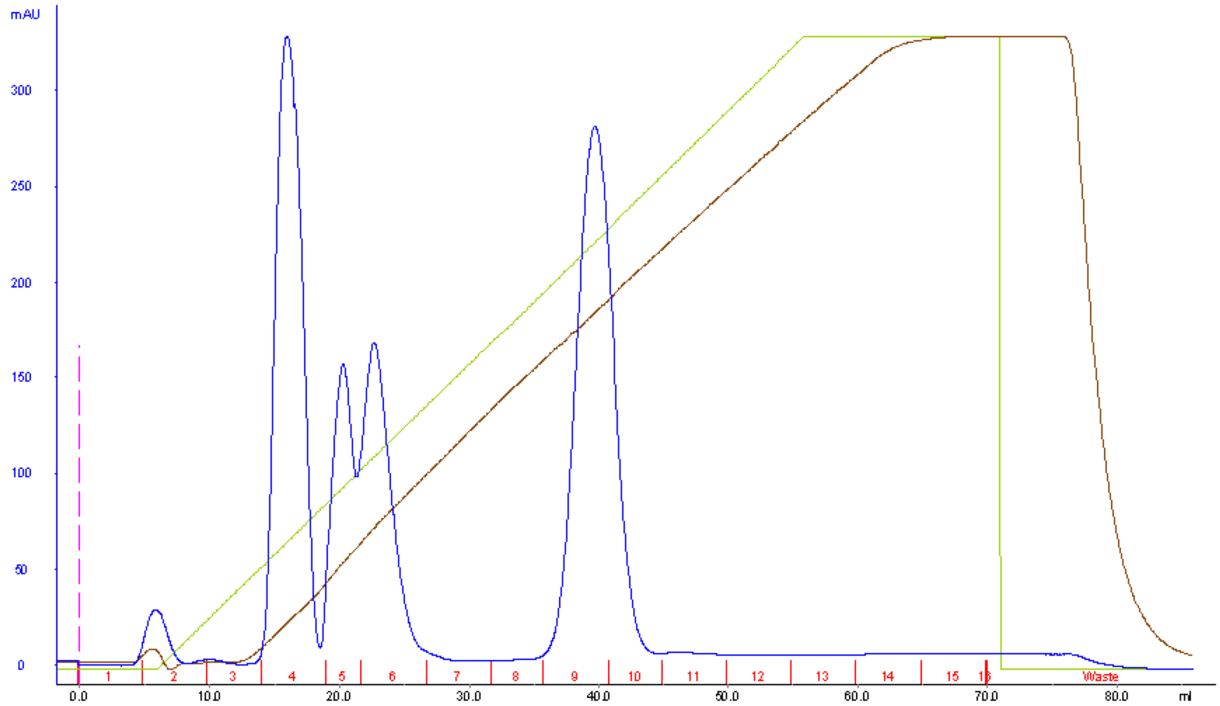

Figure S19. A representative purification of compounds from the UAXS reaction mixture used for product characterization and for evaluation of intermolecular competition experiments (blue: UV signal, brown: conductivity signal, green: concentration buffer B) is shown. Product **8** elutes in peak/fraction 4 together with UMP, product **7** elutes in peak/fraction 6, and substrate **3** elutes in peak/fraction 9-10.

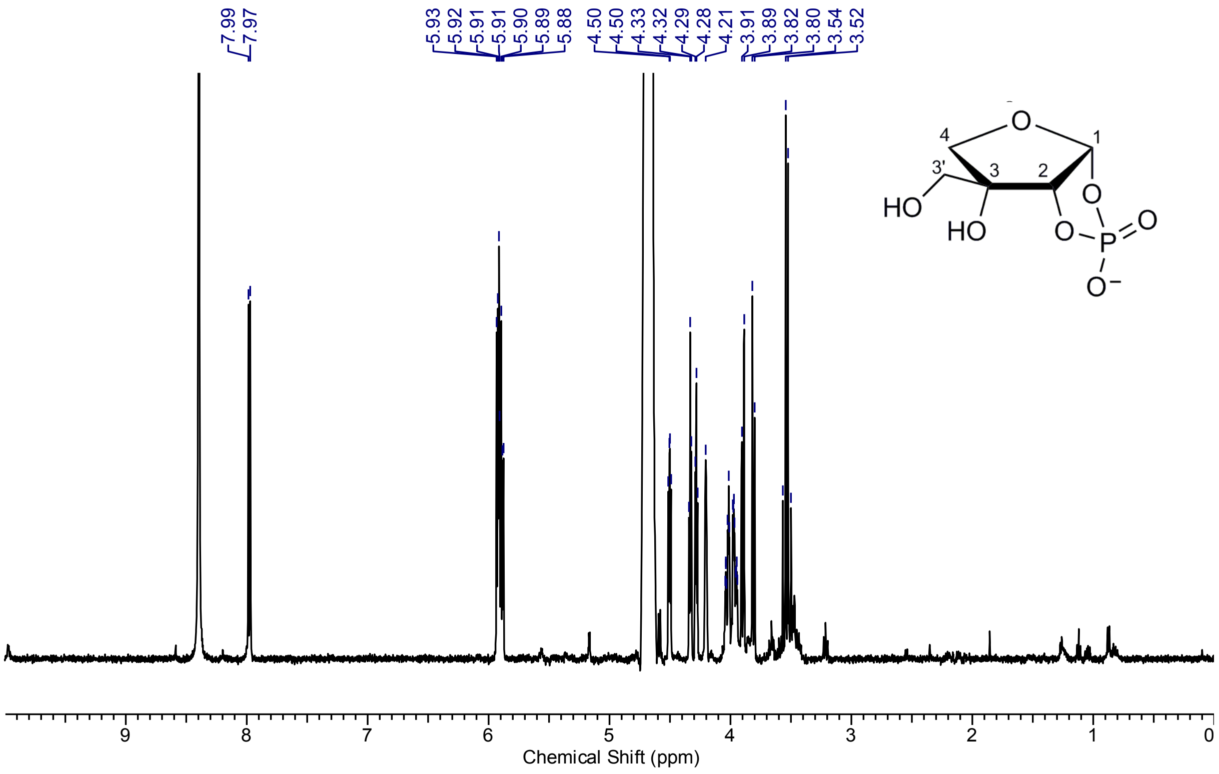

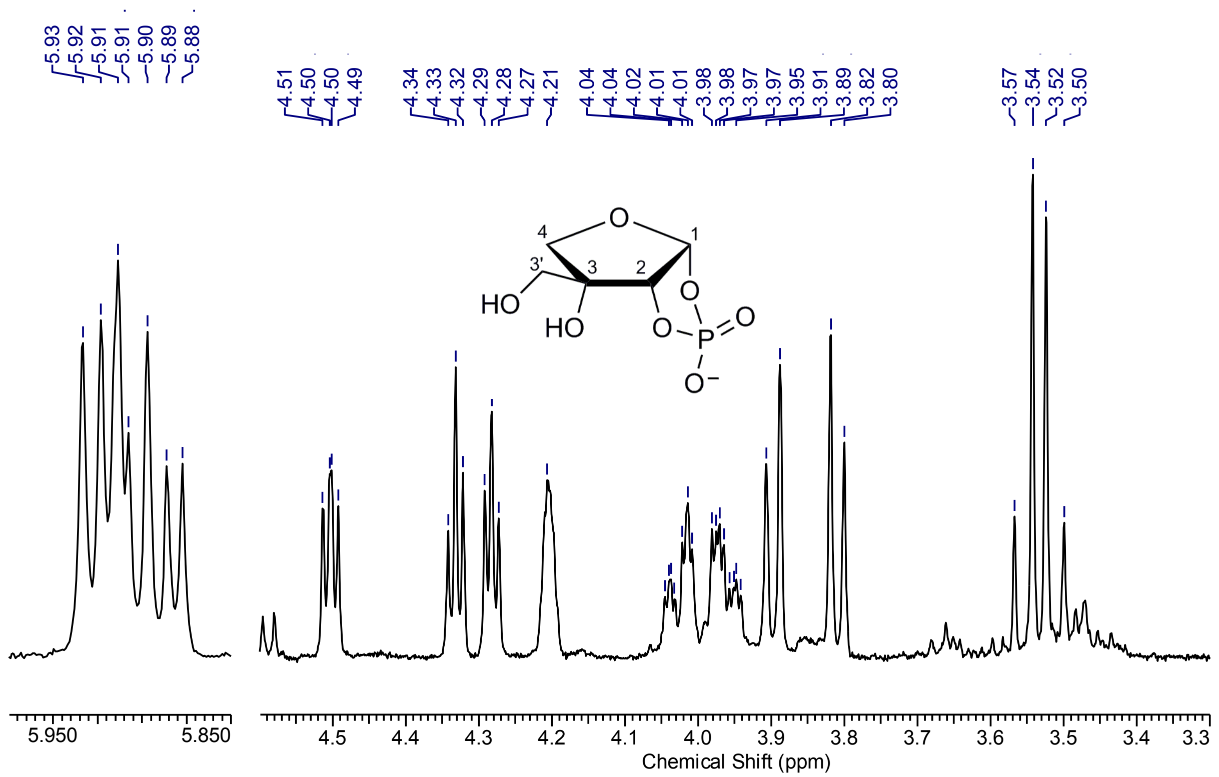

Figure S20. The cyclic phosphate **8** (containing co-eluting UMP) isolated from the UAXS reaction mixture is shown (reaction performed in H_2O).[10] The signal of *H*-1 partly overlaps with signals from UMP. ^{1}H NMR (500 MHz, D_2O) δ ppm 7.98 (d, *J*=8.30 Hz, 1 H, UMP signal), 5.86 - 5.95 (m, 3 H, UMP signals/cAP H-1), 4.50 (dd, *J*=6.10, 4.64 Hz, 1 H, cAP H-2), 4.33 (t, *J*=4.88 Hz, 1 H, UMP signal), 4.28 (t, *J*=4.88 Hz, 1 H, UMP signal), 4.21 (br. s., 1 H, UMP signal), 3.94 - 4.05 (m, 2 H, UMP signals), 3.90 (d, *J*=9.28 Hz, 1 H, cAP H-4), 3.81 (d, *J*=9.28 Hz, 1 H, cAP H-4), 3.55 (d, *J*=12.69 Hz, 1 H, cAP H-3), 3.51 (d, *J*=12.69 Hz, 1 H, cAP H-3)

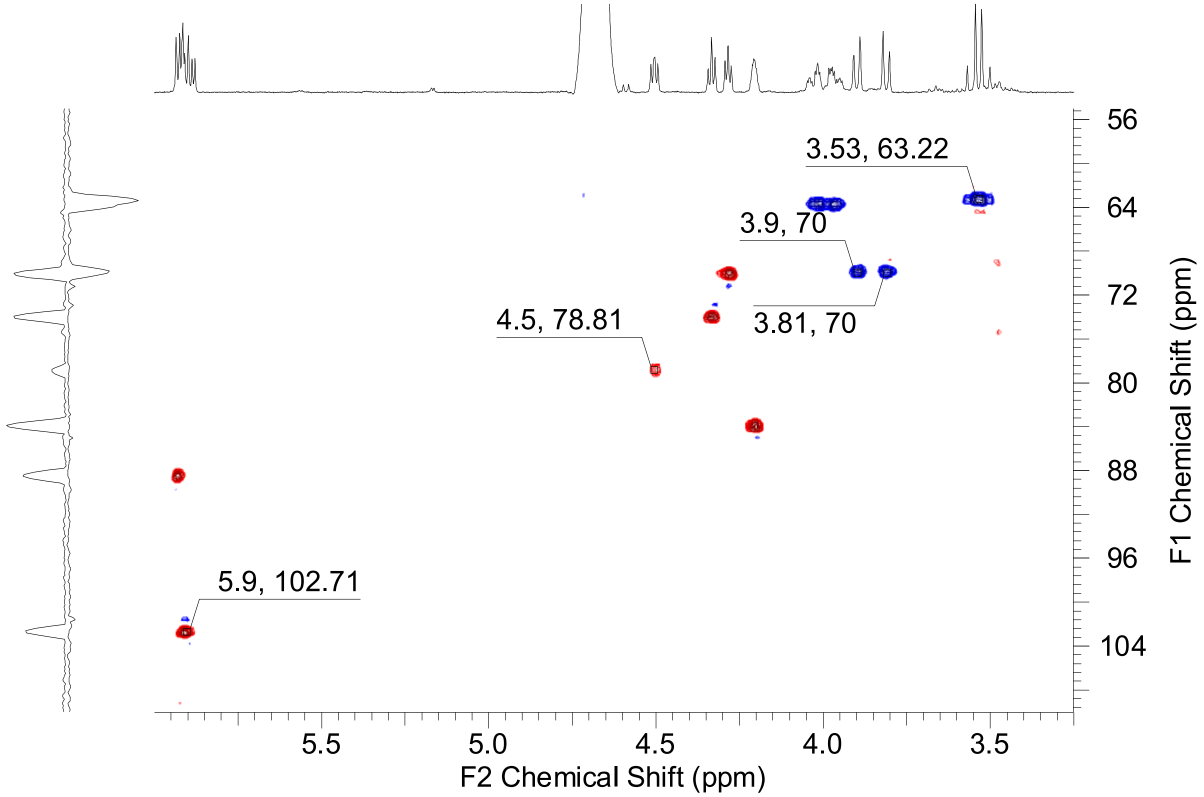

Figure S21. HSQC (F1 axis = ^{13}C, F2 axis = ^{1}H) of cyclic phosphate **8** (containing co-eluting UMP) is shown (cf. Figure S20). Signals indicated are from position 1 (102.71, 5.9) position 2 (78.81, 4.5), position 3' (63.22, 3.53) and position 4 (70, 3.81 and 70, 3.9).[10]

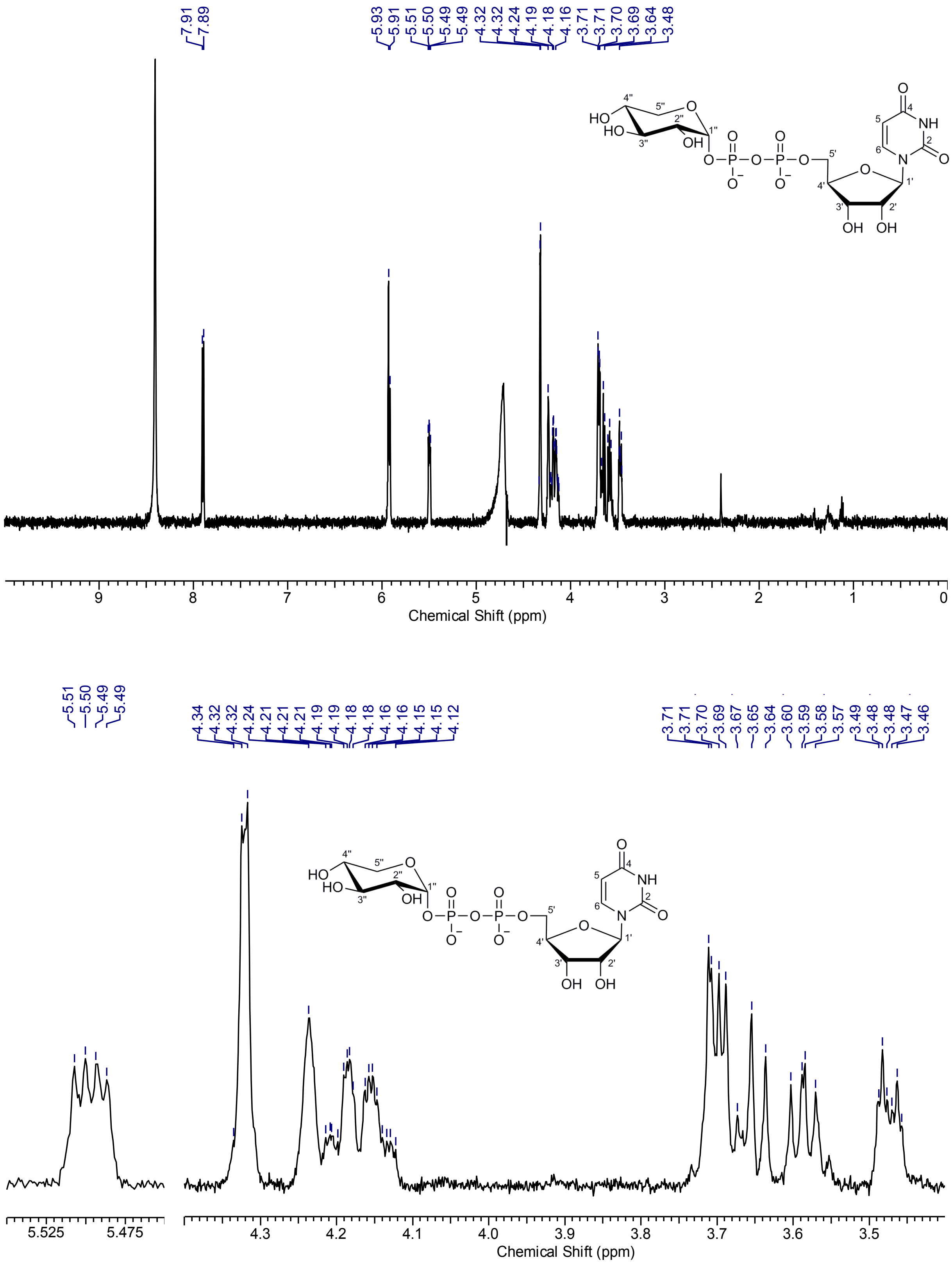

Figure S22. The reaction product **7** isolated from the UAXS reaction mixture is shown (reaction performed in H_2O).[11] ^{1}H NMR (500 MHz, D_2O) δ ppm 7.90 (d, *J*=8.30 Hz, 1 H, Ura H-6), 5.90 - 5.95 (m, 2 H, Ura H-5/Rib H-1'), 5.50 (dd, *J*=6.83, 3.42 Hz, 1 H, Xyl H-1''), 4.30 - 4.35 (m, 2 H, Rib H-2'/Rib H-3'), 4.24 (br. s., 1 H, Rib H-4'), 4.12 - 4.22 (m, 2 H, Rib H-5'), 3.68 - 3.73 (m, 2 H, Xyl H-5''), 3.65 (t, *J*=9.28 Hz, 1 H, Xyl H-3''), 3.59 (dd, *J*=9.03, 7.08 Hz, 1 H, Xyl H-4''), 3.47 (dt, *J*=9.52, 2.81 Hz, 1 H, Xyl H-2'')

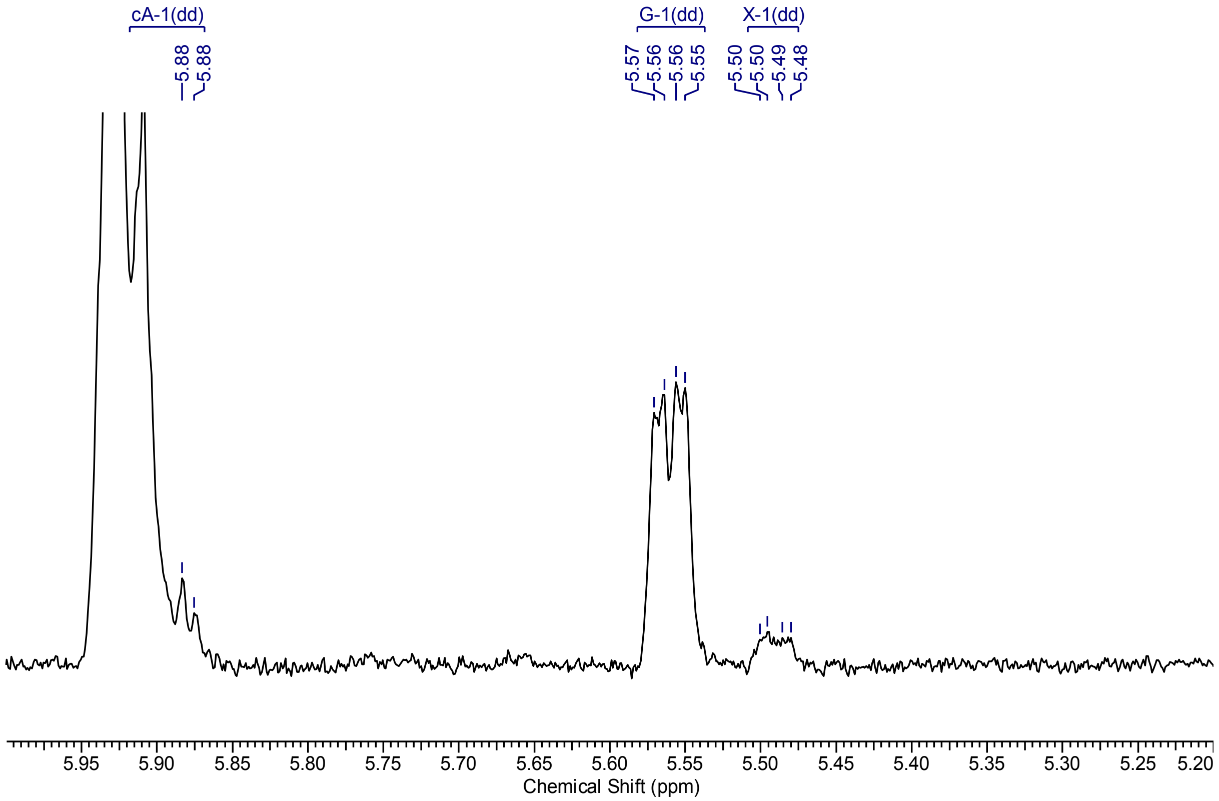

Figure S23. A representative part of the ^{1}H NMR spectrum after conversion of the [2-^{13}*C*]-**3** by UAXS in D_2O is shown. Signals of the anomeric protons from substrate (G-1), product **8** (cA-1) and product **7** (X-1) are indicated.[10]

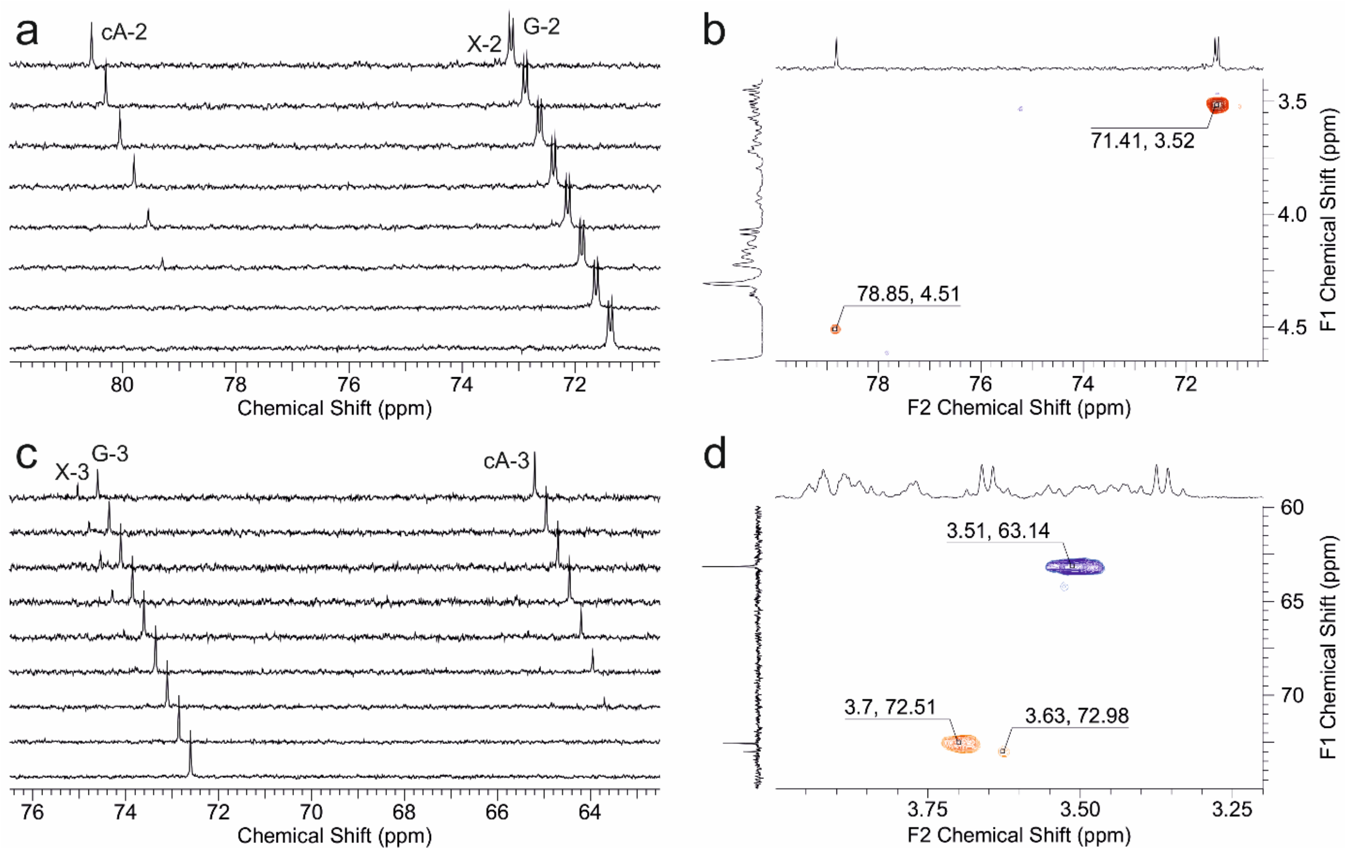

Figure S24. Results of reaction course analysis with ^{13}*C*-labeled substrates are shown (reactions performed in D_2O). **a)** In situ ^{13}C NMR measurement of 2-^{13}*C* substrate **3** conversion. Signals belonging to substrate **3** (G), product **8** (cA) and product **7** (X) are shown.[10] Time between two spectra is 150 min. **b)** HETCOR spectrum of the reaction mixture with 2-^{13}*C* substrate **3** (F1 axis = ^{1}H, F2 axis = ^{13}C). The signal at 78.85, 4.51 ppm belongs to product **8** and shows binding of the ^{13}*C* label and *H*-2 of the compound. The signal at 71.41, 3.53 ppm belongs to the substrate. **c)** In situ ^{13}C NMR measurement of 3-^{13}*C* substrate **3** conversion. Signals belonging to substrate **3** (G), product **8** (cA) and product **7** (X) are shown. Time between two spectra is 150 min. **d)** HSQC spectrum of the reaction mixture with 3-^{13}*C* substrate **2** (F1 axis = ^{13}C, F2 axis = ^{1}H; blue = negative phase, orange = positive phase). The signal at 3.51, 63.14 ppm belongs to product **8** and shows binding of the ^{13}*C* label and *H*-3 of the compound. Binding between the ^{13}*C* label and *H*-3 of product **7** is confirmed by the signal at 3.63, 72.98 ppm. The signal at 3.70, 72.51 ppm belongs to the substrate.

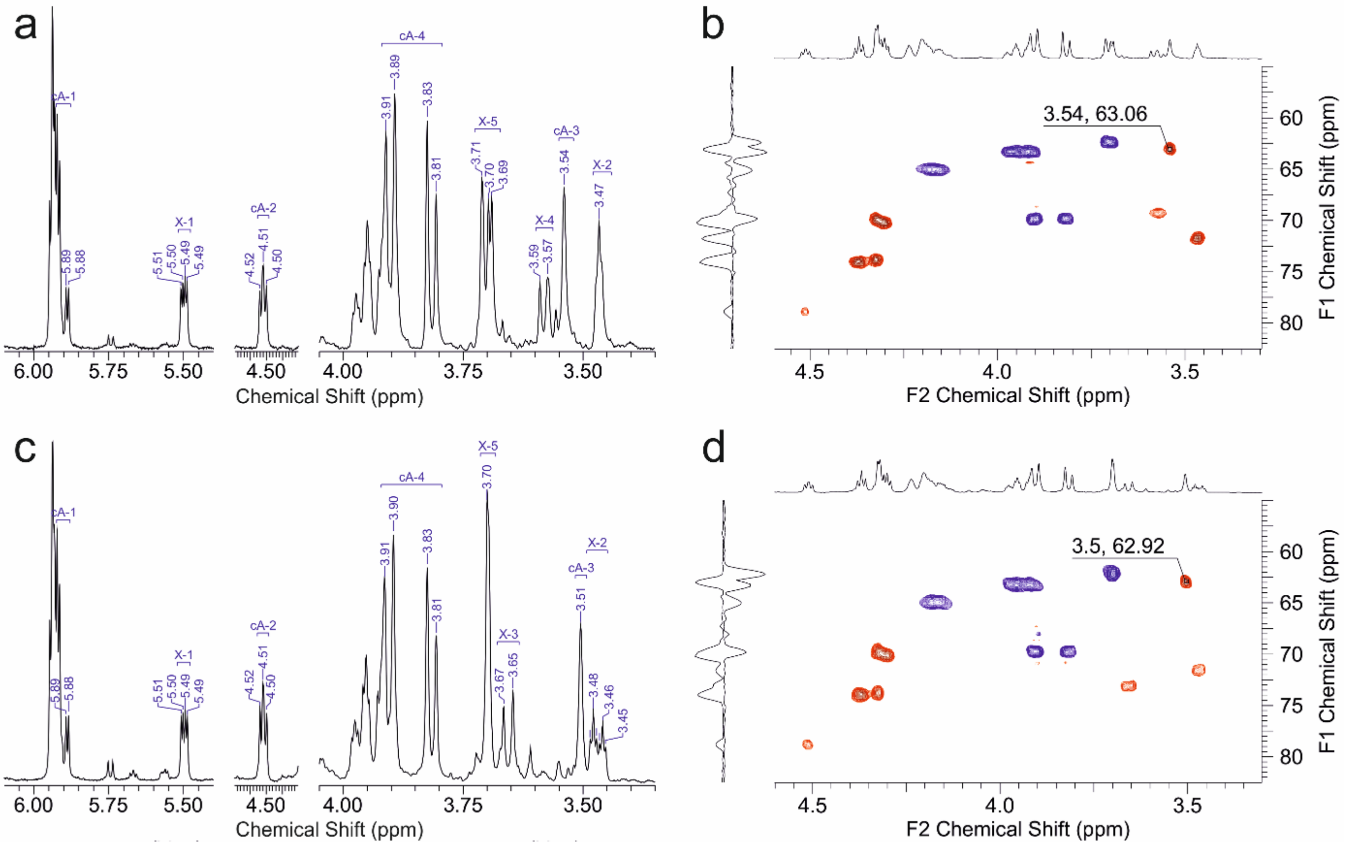

Figure S25. Results of reaction course analysis with ^{2}H-labeled substrates (reactions performed in H_2O). **a)** ^{1}H NMR spectrum after conversion of 3-^{2}H substrate **3**. Signals belonging to substrate **3** (G), product **8** (cA) and product **7** (X) are shown. Only one proton is stereospecifically exchanged by deuterium originating from position 3 of substrate **2**. **b)** HSQC spectrum of the reaction mixture shown in panel a (F1 axis = ^{13}C, F2 axis = ^{1}H; blue = negative phase, orange = positive phase). The signal at 3.54, 63.06 ppm belongs to product **8** and shows binding of the ^{2}H label and *C*-3’ of the compound. **c)** ^{1}H NMR spectrum after conversion of 4-^{2}H substrate **3**. Signals belonging to substrate **3** (G), product **8** (cA) and product **7** (X) are shown. Only one proton is stereospecifically exchanged by deuterium originating from position 4 of substrate **3**. **d)** HSQC spectrum of the reaction mixture shown in panel c (F1 axis = ^{13}C, F2 axis = ^{1}H; blue = negative phase, orange = positive phase). The signal at 3.50, 62.92 ppm belongs to product **8** and shows binding of the ^{2}H label and *C*-3’ of the compound.

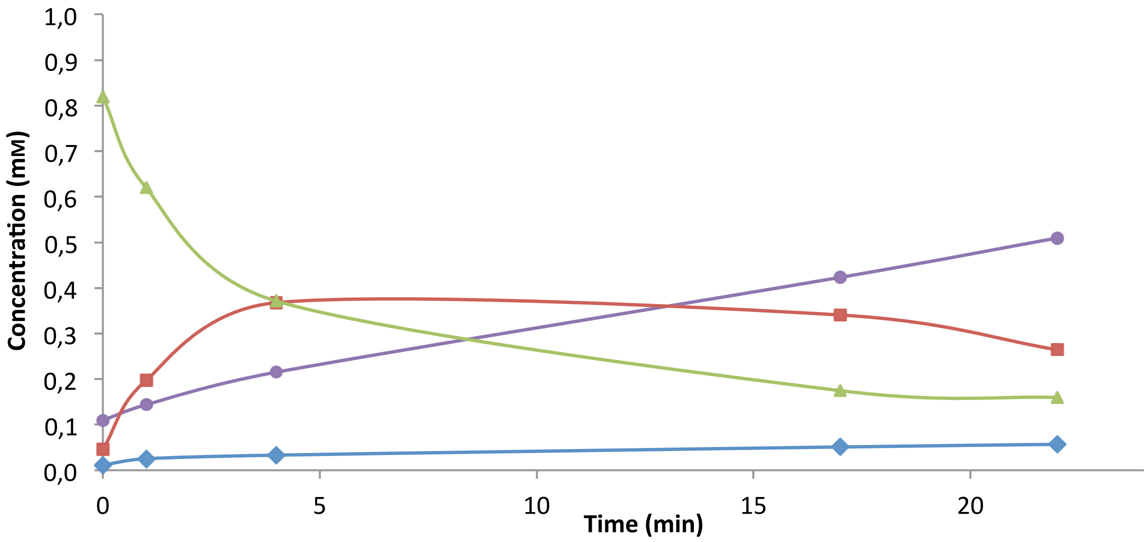

Figure S26. The conversion of substrate analogue 2-deoxy-**3** by the human UXS is shown (green: 2-deoxy-**3**, red: 2-deoxy-**7** product, blue: UMP, purple: UDP).

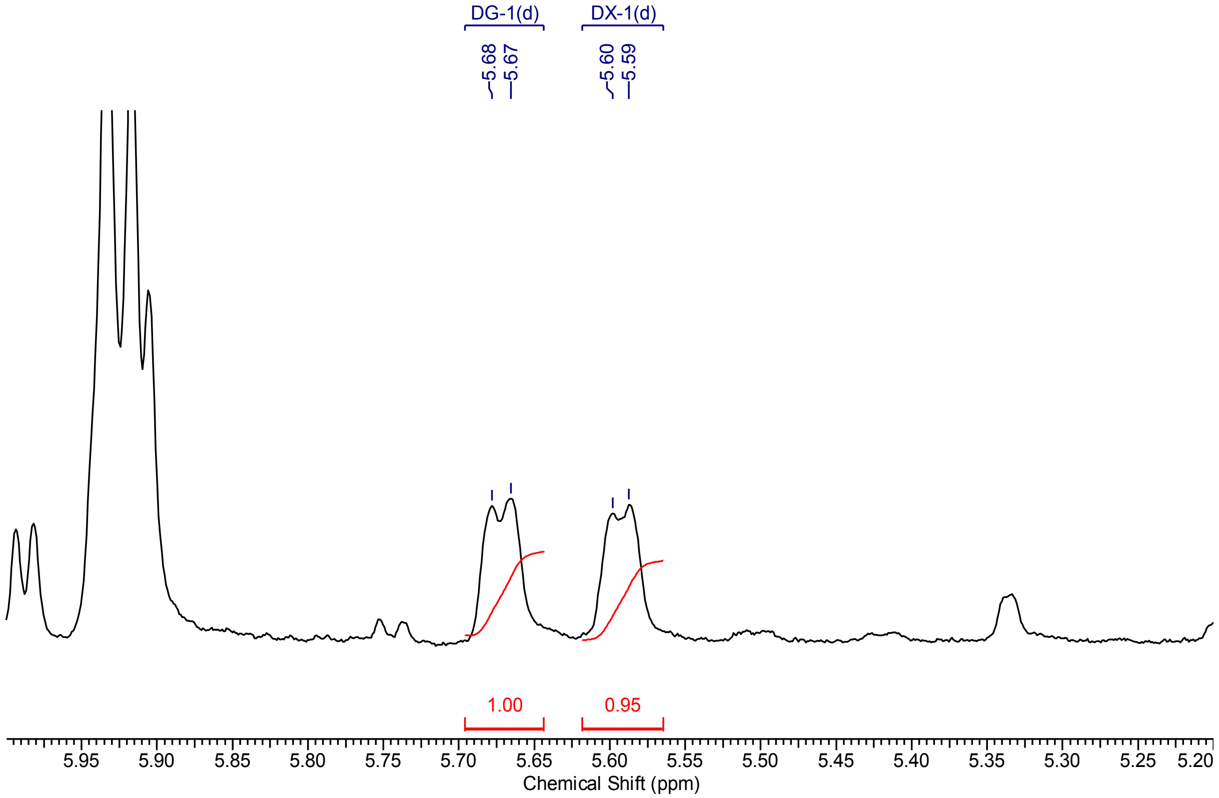

Figure S27. A representative part of the ^{1}H NMR spectrum after conversion of 2-deoxy-**3** by UXS is shown. Signals of the anomeric protons from substrate 2-deoxy-**3** (DG-1) and product 2-deoxy-**7** (DX-1) are indicated.

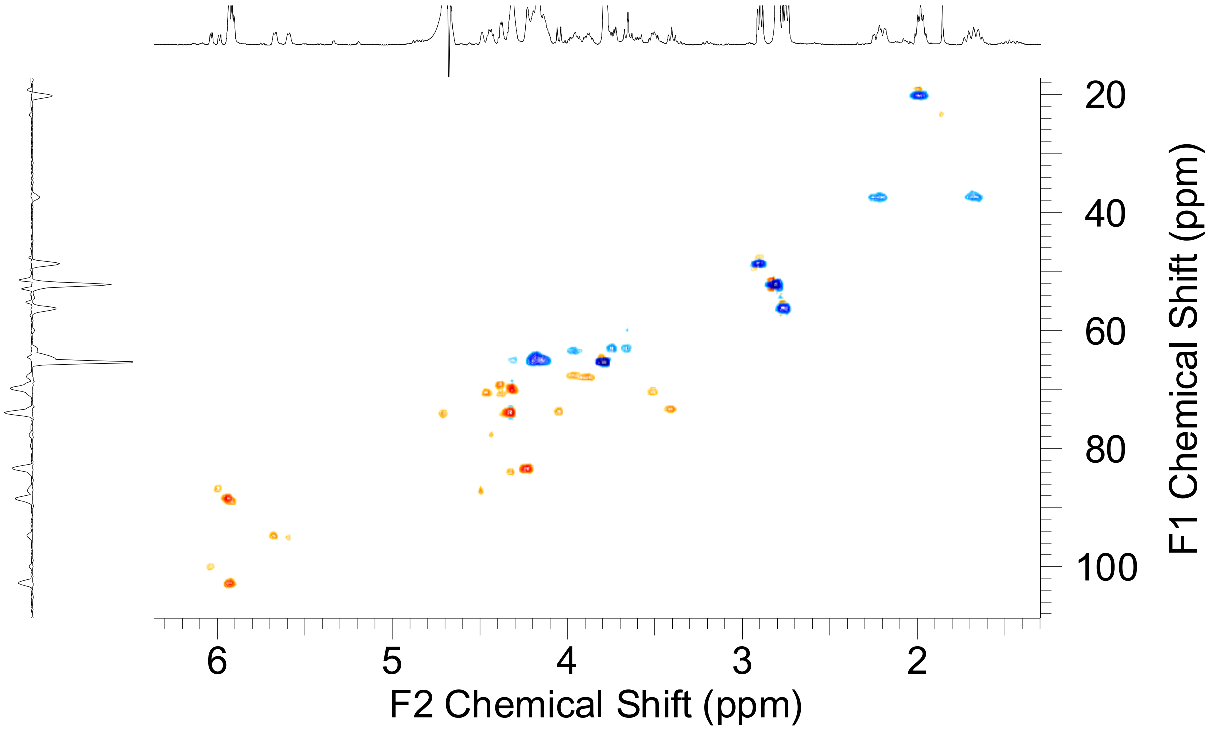

Figure S28. The HSQC spectrum (F1 axis = ^{13}C, F2 axis = ^{1}H) of reaction mixture containing substrate 2-deoxy-**3**, NAD^{+} and human UXS is shown. The formation of product 2-deoxy-**7** is visible.

DNA sequences

```
CLUSTAL O(1.2.1) multiple sequence alignment

GenBank             ---ATGGCGAATGGAGCTAATAGAGTGGATCTCGACGGGAAACCGATACAACCGTTGACA 57
Synthetic_gene      CATATGGCAAATGGTGCGAACCGTGTGGACCTGGACGGCAAACCGATTCAACCGCTGACG 60
                       ***** ***** ** **  * ***** ** ***** ******** ****** ****

GenBank             ATATGCATGATCGGCGCCGGAGGTTTCATCGGTTCACATCTCTGTGAAAAGCTCTTGACC 117
Synthetic_gene      ATCTGTATGATTGGTGCTGGCGGCTTTATTGGTAGTCATCTGTGCGAAAAACTGCTGACC 120
                    ** ** ***** ** ** ** ** ** ** ***   ***** ** ***** **  *****

GenBank             GAGACGCCACATAAGGTGCTTGCGCTCGATGTTTACAACGATAAGATCAAACACTTGCTT 177
Synthetic_gene      GAAACGCCGCACAAAGTTCTGGCCCTGGATGTCTATAACGACAAAATCAAACATCTGCTG 180
                    ** ***** ** ** ** ** ** ** ***** ** ***** ** ********  ****

GenBank             GAGCCTGATACCGTTGAATGGAGTGGTCGGATCCAGTTTCATCGTATCAATATTAAGCAT 237
Synthetic_gene      GAACCGGATACCGTTGAATGGAGCGGCCGTATTCAGTTTCATCGCATTAACATCAAACAC 240
                    ** ** ***************** ** ** ** *********** ** ** ** ** **

GenBank             GATTCGAGACTTGAAGGTCTTGTTAAGATGGCGGATCTGATTATAAATCTTGCTGCGATC 297
Synthetic_gene      GATTCTCGCCTGGAAGGTCTGGTCAAAATGGCAGACCTGATTATCAATCTGGCGGCCATT 300
                    *****  * ** ******** ** ** ***** ** ******** ***** ** ** **

GenBank             TGTACTCCAGCTGATTACAATACACGTCCTCTTGATACTATCTACAGCAATTTCATTGAT 357
Synthetic_gene      TGTACGCCGGCTGATTATAACACCCGTCCGCTGGACACGATCTACAGTAATTTCATTGAT 360
                    ***** ** ******** ** ** ***** ** ** ** ******** ************

GenBank             GCGCTTCCAGTTGTGAAATACTGTTCTGAGAACAACAAGCGTCTCATTCACTTTTCTACC 417
Synthetic_gene      GCACTGCCGGTGGTTAAATACTGCAGTGAAAACAACAAACGCCTGATCCATTTTTCCACC 420
                    ** ** ** ** ** ********   *** ******** ** ** ** ** ***** ***

GenBank             TGTGAAGTTTATGGAAAAACCATTGGAAGCTTTCTTCCTAAGGATCATCCTCTGCGTGAT 477
Synthetic_gene      TGTGAAGTGTACGGCAAAACGATTGGTTCCTTCCTGCCGAAAGATCACCCGCTGCGTGAT 480
                    ******** ** ** ***** *****   *** ** ** ** ***** ** *********

GenBank             GATCCTGCTTTCTATGTTCTTAAAGAAGATATTTCCCCTTGCATATTTGGTTCAATTGAG 537
Synthetic_gene      GACCCGGCTTTTTATGTTCTGAAAGAAGACATTAGTCCGTGCATCTTCGGCTCCATTGAA 540
                    ** ** ***** ******** ******** ***   ** ***** ** ** ** *****

GenBank             AAGCAGAGGTGGTCATATGCGTGTGCAAAGCAACTGATTGAGAGACTTGTTTACGCTGAG 597
Synthetic_gene      AAACAGCGTTGGTCTTATGCATGTGCTAAACAACTGATTGAACGCCTGGTCTACGCGGAA 600
                    ** *** * ***** ***** ***** ** ***********  * ** ** ***** **

GenBank             GGTGCTGAGAATGGGCTTGAGTTCACCATCGTACGACCTTTTAACTGGATTGGACCTAGG 657
Synthetic_gene      GGCGCCGAAAACGGTCTGGAATTTACCATCGTGCGTCCGTTCAATTGGATTGGCCCGCGC 660
                    ** ** ** ** ** ** ** ** ******** ** ** ** ** ******** **  *

GenBank             ATGGATTTCATCCCCGGCATTGATGGTCCTAGCGAAGGTGTCCCACGTGTCCTTGCCTGC 717
Synthetic_gene      ATGGATTTTATTCCGGGCATCGACGGTCCGTCAGAAGGTGTGCCGCGCGTTCTGGCGTGC 720
                    ******** ** ** ***** ** *****    ******** ** ** ** ** ** ***

GenBank             TTTAGTAACAATCTTCTACGTCGTGAGCCTCTCAAGCTTGTAGATGGTGGAGAATCACAG 777
Synthetic_gene      TTCTCGAACAATCTGCTGCGTCGCGAACCGCTGAAACTGGTGGATGGCGGTGAAAGCCAG 780
                    **    ******** ** ***** ** ** ** ** ** ** ***** ** ***   ***

GenBank             AGAACTTTCGTCTACATCAATGATGCTATTGAAGCTGTCCTTTTGATGATTGAAAACCCA 837
Synthetic_gene      CGTACCTTTGTGTATATCAATGACGCAATTGAAGCTGTTCTGCTGATGATCGAAAACCCG 840
                     * ** ** ** ** ******** ** *********** **  ******* ********

GenBank             GAGAGGGCAAATGGGCACATCTTCAACGTAGGCAACCCGAACAACGAAGTTACAGTAAGA 897
Synthetic_gene      GAACGTGCGAATGGCCACATTTTCAACGTTGGTAATCCGAACAATGAAGTCACCGTGCGC 900
                    **  * ** ***** ***** ******** ** ** ******** ***** ** **  *

GenBank             CAGCTCGCTGAAATGATGACCGAGGTTTACGCAAAAGTGAGTGGAGAAGGAGCCATTGAG 957
Synthetic_gene      CAACTGGCCGAAATGATGACGGAAGTCTACGCGAAAGTGTCAGGCGAAGGTGCCATCGAA 960
                    ** ** ** *********** ** ** ***** ******   ** ***** ***** **

GenBank             AGCCCAACGGTTGATGTTAGCTCCAAAGAGTTTTACGGGGAAGGTTATGATGACAGTGAC 1017
Synthetic_gene      TCGCCGACCGTTGATGTCAGCTCTAAAGAATTTTATGGCGAAGGTTACGATGACAGCGAT 1020
                       ** ** ******** ***** ***** ***** ** ******** ******** **
```

```
GenBank             AAGAGAATCCCAGACATGACCATCATTAACCGCCAACTCGGATGGAACCCGAAAACATCG 1077
Synthetic_gene      AAACGTATTCCGGACATGACCATTATCAACCGCCAACTGGGTTGGAATCCGAAAACGAGC 1080
                    **  * ** ** *********** ** *********** ** ***** ********

GenBank             CTATGGGACTTGCTCGAGTCGACCTTAACCTACCAGCACAGGACATACGCTGAAGCTGTG 1137
Synthetic_gene      CTGTGGGATCTGCTGGAATCTACCCTGACGTATCAACATCGCACCTATGCCGAAGCAGTG 1140
                    ** *****  **** ** ** *** * ** ** ** **  * ** ** ** ***** ***

GenBank             AAGAAGGCAACATCCAAACCAGTGGCTTCCTAA---       1170
Synthetic_gene      AAAAAAGCTACCAGCAAACCGGTCGCAAGCCTCGAG       1176
                    ** ** ** **   ****** ** **   *
```

Figure S29. Sequence comparison of original AXS1 gene from GenBank and codon-optimized synthetic AXS1 gene used for recombinant UAXS production.

```
CLUSTAL O(1.2.1) multiple sequence alignment

Synthetic_gene      -----------------------------CATATGGCAAATGGTGCGAACCGTGTGGACC 31
Sequenced_gene      ATTTTGTTTAACTTTAAGAAGGAGATATACATATGGCAAATGGTGCGAACCGTGTGGACC 60
                                                 *******************************

Synthetic_gene      TGGACGGCAAACCGATTCAACCGCTGACGATCTGTATGATTGGTGCTGGCGGCTTTATTG 91
Sequenced_gene      TGGACGGCAAACCGATTCAACCGCTGACGATCTGTATGATTGGTGCTGGCGGCTTTATTG 120
                    ************************************************************

Synthetic_gene      GTAGTCATCTGTGCGAAAAACTGCTGACCGAAACGCCGCACAAAGTTCTGGCCCTGGATG 151
Sequenced_gene      GTAGTCATCTGTGCGAAAAACTGCTGACCGAAACGCCGCACAAAGTTCTGGCCCTGGATG 180
                    ************************************************************

Synthetic_gene      TCTATAACGACAAAATCAAACATCTGCTGGAACCGGATACCGTTGAATGGAGCGGCCGTA 211
Sequenced_gene      TCTATAACGACAAAATCAAACATCTGCTGGAACCGGATACCGTTGAATGGAGCGGCCGTA 240
                    ************************************************************

Synthetic_gene      TTCAGTTTCATCGCATTAACATCAAACACGATTCTCGCCTGGAAGGTCTGGTCAAAATGG 271
Sequenced_gene      TTCAGTTTCATCGCATTAACATCAAACACGATTCTCGCCTGGAAGGTCTGGTCAAAATGG 300
                    ************************************************************

Synthetic_gene      CAGACCTGATTATCAATCTGGCGGCCATTTGTACGCCGGCTGATTATAACACCCGTCCGC 331
Sequenced_gene      CAGACCTGATTATCAATCTGGCGGCCATTTGTACGCCGGCTGATTATAACACCCGTCCGC 360
                    ************************************************************

Synthetic_gene      TGGACACGATCTACAGTAATTTCATTGATGCACTGCCGGTGGTTAAATACTGCAGTGAAA 391
Sequenced_gene      TGGACACGATCTACAGTAATTTCATTGATGCACTGCCGGTGGTTAAATACTGCAGTGAAA 420
                    ************************************************************

Synthetic_gene      ACAACAAACGCCTGATCCATTTTTCCACCTGTGAAGTGTACGGCAAAACGATTGGTTCCT 451
Sequenced_gene      ACAACAAACGCCTGATCCATTTTTCCACCTGTGAAGTGTACGGCAAAACGATTGGTTCCT 480
                    ************************************************************

Synthetic_gene      TCCTGCCGAAAGATCACCCGCTGCGTGATGACCCGGCTTTTTATGTTCTGAAAGAAGACA 511
Sequenced_gene      TCCTGCCGAAAGATCACCCGCTGCGTGATGACCCGGCTTTTTATGTTCTGAAAGAAGACA 540
                    ************************************************************

Synthetic_gene      TTAGTCCGTGCATCTTCGGCTCCATTGAAAAACAGCGTTGGTCTTATGCATGTGCTAAAC 571
Sequenced_gene      TTAGTCCGTGCATCTTCGGCTCCATTGAAAAACAGCGTTGGTCTTATGCATGTGCTAAAC 600
                    ************************************************************

Synthetic_gene      AACTGATTGAACGCCTGGTCTACGCGGAAGGCGCCGAAAACGGTCTGGAATTTACCATCG 631
Sequenced_gene      AACTGATTGAACGCCTGGTCTACGCGGAAGGCGCCGAAAACGGTCTGGAATTTACCATCG 660
                    ************************************************************

Synthetic_gene      TGCGTCCGTTCAATTGGATTGGCCCGCGCATGGATTTTATTCCGGGCATCGACGGTCCGT 691
Sequenced_gene      TGCGTCCGTTCAATTGGATTGGCCCGCGCATGGATTTTATTCCGGGCATCGACGGTCCGT 720
                    ************************************************************

Synthetic_gene      CAGAAGGTGTGCCGCGCGTTCTGGCGTGCTTCTCGAACAATCTGCTGCGTCGCGAACCGC 751
Sequenced_gene      CAGAAGGTGTGCCGCGCGTTCTGGCGTGCTTCTCGAACAATCTGCTGCGTCGCGAACCGC 780
                    ************************************************************
```

```
Synthetic_gene      TGAAACTGGTGGATGGCGGTGAAAGCCAGCGTACCTTTGTGTATATCAATGACGCAATTG 811
Sequenced_gene      TGAAACTGGTGGATGGCGGTGAAAGCCAGCGTACCTTTGTGTATATCAATGACGCAATTG 840
                    ************************************************************

Synthetic_gene      AAGCTGTTCTGCTGATGATCGAAAACCCGGAACGTGCGAATGGCCACATTTTCAACGTTG 871
Sequenced_gene      AAGCTGTTCTGCTGATGATCGAAAACCCGGAACGTGCGAATGGCCACATTTTCAACGTTG 900
                    ************************************************************

Synthetic_gene      GTAATCCGAACAATGAAGTCACCGTGCGCCAACTGGCCGAAATGATGACGGAAGTCTACG 931
Sequenced_gene      GTAATCCGAACAATGAAGTCACCGTGCGCCAACTGGCCGAAATGATGACGGAAGTCTACG 960
                    ************************************************************

Synthetic_gene      CGAAAGTGTCAGGCGAAGGTGCCATCGAATCGCCGACCGTTGATGTCAGCTCTAAAGAAT 991
Sequenced_gene      CGAAAGTGTCAGGCGAAGGTGCCATCGAATCGCCGACCGTTGATGTCAGCTCTAAAGAAT 1020
                    ************************************************************

Synthetic_gene      TTTATGGCGAAGGTTACGATGACAGCGATAAACGTATTCCGGACATGACCATTATCAACC 1051
Sequenced_gene      TTTATGGCGAAGGTTACGATGACAGCGATAAACGTATTCCGGACATGACCATTATCAACC 1080
                    ************************************************************

Synthetic_gene      GCCAACTGGGTTGGAATCCGAAAACGAGCCTGTGGGATCTGCTGGAATCTACCCTGACGT 1111
Sequenced_gene      GCCAACTGGGTTGGAATCCGAAAACGAGCCTGTGGGATCTGCTGGAATCTACCCTGACGT 1140
                    ************************************************************

Synthetic_gene      ATCAACATCGCACCTATGCCGAAGCAGTGAAAAAAGCTACCAGCAAACCGGTCGCAAGCC 1171
Sequenced_gene      ATCAACATCGCACCTATGCCGAAGCAGTGAAAAAAGCTACCAGCAAACCGGTCGCAAGCC 1200
                    ************************************************************

Synthetic_gene      TCGAG------------------------------------------     1176
Sequenced_gene      TCGAGCACCACCACCACCACCACTGAGATCCGGCTGCTAACAAAGCC     1247
                    *****
```

Figure S30. Sequence comparison of synthetic AXS1 gene as obtained by the supplier and sequencing results after plasmid isolation. The *C*-terminal His_6 tag is visible at the end of the sequenced gene.

References

(1) Egger, S.; Chaikuad, A.; Kavanagh, K. L.; Oppermann, U.; Nidetzky, B. *J. Biol. Chem.* **2011**, *286*, 23877-23887.

(2) Pival, S. L.; Klimacek, M.; Kratzer, R.; Nidetzky, B. *FEBS Lett.* **2008**, *582*, 4095-4099.

(3) Wildberger, P.; Brecker, L.; Nidetzky, B. *Carbohydr. Res.* **2012**, *356*, 224-232.

(4) Eixelsberger, T.; Sykora, S.; Egger, S.; Brunsteiner, M.; Kavanagh, K. L.; Oppermann, U.; Brecker, L.; Nidetzky, B. *J. Biol. Chem.* **2012**, *287*, 31349-31358.

(5) Lee, S. S.; Hong, S. Y.; Errey, J. C.; Izumi, A.; Davies, G. J.; Davis, B. G. *Nat. Chem. Biol.* **2011**, *7*, 631-638.

(6) Ma, X.; Stöckigt, J. *Carbohydr. Res.* **2001**, *333*, 159-163.

(7) Eixelsberger, T.; Nidetzky, B. *Adv. Synth. Catal.* **2014**, *356*, 3575-3584.

(8) Cavaluzzi, M. J.; Borer, P. N. *Nucleic Acids Res.* **2004**, *32*, e13.

(9) Crich, D.; Chandrasekera, N. S. *Angew. Chem. Int. Ed.* **2004**, *43*, 5386-5389.

(10) Guyett, P.; Glushka, J.; Gu, X.; Bar-Peled, M. *Carbohydr. Res.* **2009**, *344*, 1072-1078.

(11) Bar-Peled, M.; Griffith, C. L.; Doering, T. L. *Proc. Natl. Acad. Sci. U.S.A* **2001**, *98*, 12003-12008.
